# From dysbiosis to longevity: a narrative review into the gut microbiome’s impact on aging

**DOI:** 10.1186/s12929-025-01179-x

**Published:** 2025-10-11

**Authors:** Ching-Hung Tseng, Chun-Ying Wu

**Affiliations:** 1 Germark Biotechnology Co., Ltd, Taichung, 407754 Taiwan; 2 Institute of Biomedical Informatics, National Yang Ming Chiao Tung University, Taipei, 112304 Taiwan; 3 Microbiota Research Center, National Yang Ming Chiao Tung University, Taipei, 112304 Taiwan; 4 Health Innovation Center, National Yang Ming Chiao Tung University, Taipei, 112304 Taiwan; 5 Ph.D. Program of Interdisciplinary Medicine, National Yang Ming Chiao Tung University, Taipei, 112304 Taiwan; 6 Division of Translational Research, Taipei Veterans General Hospital, Taipei, 112201 Taiwan; 7 Department of Medical Research, Taichung Veterans General Hospital, Taichung, 407219 Taiwan; 8 College of Public Health, China Medical University, Taichung, 406040 Taiwan

**Keywords:** Gut microbiome, Healthy aging, Longevity, Eubionts, Pathobionts, Gut barrier, Inflammation, Gut-muscle axis, Gut-brain axis

## Abstract

Aging has become an important public health concern with the accelerated aging of the global population. The rising impetus to extend lifespan as well as healthspan has drawn attention to the gut microbiome, an indispensable yet modifiable determinant of the aging process. This narrative review addresses the complex interaction between the gut microbiome and aging, synthesizing findings in logical order. Evidence from model organisms supports the causal influence of gut microbes on host aging and longevity. Developmental evolution of the human gut microbiome throughout life stages reflects its adaptive nature affected by diet, lifestyle, hormone levels, and immune function, regulating aging through the gut-muscle and the gut-brain axes in late life. Signature characteristics of the long-lived gut microbiome, including increased diversity, elevated beneficial taxa, and enhanced gut homeostasis, lead to strategies to extend longevity. Intake of fiber, regular exercise, and pro-/pre-/postbiotic supplements are potential interventions on the gut microbiome to foster vitality in later years. Centering on these connected topics, this review identifies questions warranting investigation, with potential to improve therapeutic strategies for healthy aging.

## Introduction

The global population is rapidly shifting to an older age demographic [1] due to declining fertility rate and rising life expectancy [2]. Aging is an unavoidable component of biological life, during which occurs a deterioration of physiological functions and an increase in fragility, leading to greater susceptibility to infections and diseases. Together, these contribute to elevated mortality rates within the aged cohorts. Understanding the processes of aging is therefore an important research direction with significant social and scientific impacts [3], underscoring the urgency of developing effective ways to promote healthy aging. Healthy aging refers to preserving optimal physical and mental function in later life so that one can engage in daily living activities independently and maintain social participation. Healthy aging also emphasizes not just extending lifespan but increasing healthspan, the period of life when one is free of limiting chronic injuries and maintain healthy functionality, while at the same time promoting dignity and vitality in the process of aging. Healthy aging is influenced by many factors. In addition to genetic influences, which are beyond our control, modifiable factors—such as exercise, a balanced and nutritious diet, and routine medical checkups—can all contribute to prolonged physical and mental components of aging, increasing both life- and healthspan.

Aging is a complex process marked by the progressive decline of systemic health. Central to this understanding are nine hallmarks of aging, reflecting diverse molecular and cellular mechanisms that drive the aging process [4]. Additionally, emerging evidence highlights a potential tenth hallmark, the disturbance of the gut microbiome [5, 6]. The gut microbiome is a complex microbial community that plays an essential role in human health [7, 8] and has been regarded as being equivalent to a forgotten organ [9]. However, the gut microbiome might not conform to the typical pattern of decline with aging in the human body [10]. Instead, it exhibits unique dynamics associated with aging processes and has been speculatively linked to age-related comorbidities [11]. This distinct behavior underscores its significance and highlights the need for a deeper understanding of its role in health and aging.

The vast body of literature makes it impossible to address all studies exploring the intricate connections between the gut microbes and aging within a single paper. As a result, we have focused on specific topics and selected representative studies for each section, while acknowledging that some related works may not be included. The gut microbiome and its relationship with aging, recognized as a critical public health issue, has previously been extensively reviewed from various angles [12–14]. In this review, we examine key themes related to the gut microbiome and aging. These include evidence from animal models demonstrating the gut microbiome’s role in the aging process, its progression across the lifespan from infancy to adulthood and old age, the mechanisms linking the gut microbiome to aging, and potential interventions targeting the microbiome to address aging-related changes.

## Gut microbes modify the aging process in animal models

Aging is a multi-systemic process in which physiological functions progressively decline over time. Decreased gut barrier integrity and the development of chronic, low-grade inflammation, known as ‘inflammaging’, are prominent contributors to systemic aging-related health conditions [15, 16]. In this context, the gut–brain axis (associated with cognitive decline and neurodegeneration) and the gut–muscle axis (associated with frailty) are particularly relevant to host aging and, ultimately, longevity. In support of this, numerous experiments using model organisms have demonstrated a strong link between gut bacteria and host lifespan.

The influence of the gut microbiome on the aging process has been documented in vertebrate models. Using the naturally short-lived African turquoise killifish, transferring the gut microbiome from young to middle-aged killifish resulted in a prolonged lifespan and delayed behavioral decline when compared to those receiving a microbiome transplant from the same age-cohort middle-aged killifish [17]. Additionally, co-housing germ-free young mice with old, conventionally raised mice led to an increase in blood pro-inflammatory cytokines in the young mice. However, this effect was absent in germ-free mice deficient in TNF-α (*tnfα*^−/−^), providing evidence that age-related microbial dysbiosis exacerbates systemic inflammation via TNF-α signaling [18]. Fecal transplantation from wild-type mice into progeroid mice reduced disease phenotypes and increased lifespan, and supplementation with *Akkermansia muciniphila* independently also increased lifespan modestly compared to controls [19]. Metabolomic analysis of these mice suggested that the mechanism for lifespan extension was generally due to the restoration of secondary bile acid synthesis through the gut microbiome remodeling. Furthermore, another study transplanting the fecal microbiome from a long-lived individual (101 years old) to eleven-month-old mice resulted in more beneficial taxa, higher microbial diversity, less lipofuscin accumulation in the brain, and longer intestinal villi in the recipients compared to those receiving transplants from an elderly donor (70 years old) [20]. More recently, transplanting the microbiome from young mice restored gut barrier integrity and reduced intestinal inflammation in older mice, with results implying the beneficial effects were achieved by maintaining gut homeostasis [21]. A recent comprehensive metabolic modeling study combining tissue transcriptome, metagenome, and metabolome data to examine aging-related systemic dynamics across five age groups of mice (ranging from 2 to 30 months old) revealed increased systemic inflammation and downregulated cellular maintenance and tissue regeneration processes in aged mice [22]. Notably, nucleotide metabolism was predicted to depend on the microbiome and was reported to partly underlie aging-associated declines in gut barrier integrity.

Studies in invertebrate models have also provided insights into the role and function of the gut microbiome in the process of aging. In *Caenorhabditis elegans*, an isolated *Escherichia coli* mutant with disrupted folate synthesis was unexpectedly found to slow *C. elegans* aging [23]. Another large-scale screening of *E. coli* mutants identified 29 genes whose deletion extended the lifespan of *C. elegans*, among which five mutants increased *E. coli* colanic acid production, thereby promoting mitochondrial dynamics and regulating unfolded protein response in the host, a process that helps maintain proteostasis under stress [24]. In addition, nitric oxide produced by commensal bacteria was shown to enhance *C. elegans* longevity [25]. Recently, the 3-phenyllactic acid produced by the probiotic *Lactiplantibacillus plantarum* effectively prolonged *C. elegans* lifespan by enhancing energy metabolism and stress resilience [26]. Together, these findings suggest that microbial genetic and metabolic variation, particularly within *E. coli*, can modulate host aging by influencing mitochondrial function, stress response, and key metabolic pathways.

In *Drosophila melanogaster*, a pioneering study found that reintroducing bacteria to axenic embryos or early-adult flies by adding a homogenate of nonaxenic flies to the food could enhance lifespan [27]. However, an independent study reported no such effect, attributing the discrepancy to differences in culture conditions [28]. This was later clarified by showing that gut bacteria can both extend or shorten fly lifespan, depending on the nutritional environment [29]. Low-dose oxidant exposure during *D. melanogaster* development extended adult longevity by reducing *Acetobacter* proteobacteria [30], whose indigenous presence was associated with age-related gut dysfunction and reduced lifespan. Although driven by different mechanisms, commensal dysbiosis resulting from aging-related immune signaling deregulation [31] and compromised gut compartmentalization [32] in fruit flies reduced their lifespan. Through a comparison of gene expression profiles in conventionally and axenically reared *D. melanogaster*, stress response and innate immunity genes represented two of the nine hallmarks of aging with expression influenced by the microbiome rather than chronological age [33].

While invertebrate models have provided important insights into the biological role of the gut microbiome in aging, translating these findings to humans remains limited due to differences in anatomical, physiological, and immune complexity. Therefore, findings from these models should be interpreted in the context of complementary evidence from vertebrate and specifically mammalian systems.

Collectively, these studies have highlighted the close connection between the gut microbiome and host aging. They have identified that microbial factors and interventions improve host longevity and slow aging by modifying several aging hallmarks, including rescuing mitochondrial dysfunction, restoring proteostasis, improving stress resistance and immune function, reducing inflammaging, enhancing gut integrity, and remodeling age-associated gut dysbiosis (Table 1). With the ultimate goal of promoting healthy human aging, animal studies have revealed several intervention options, such as fecal transplantation and supplementing metabolites or commensal species. As more microbiome-dependent pathways are identified, the targeted design of strategies to enhance pathway activities by microbiome remodeling also paves the way for advancing healthy aging approaches.

**Table 1:** Evidence list of microbial factors or interventions shown to improve host longevity and aging hallmarks in model organisms

Model organism	Microbial factor or intervention	Observed effect/mechanisms	Corresponding modifications on aging hallmark(s)	References
Killifish	Fecal microbiota transplant from young to middle-aged fish	Extended lifespan and delayed behavioral decline/enhanced defense to bacteria, Tor pathway, and extracellular matrix by young microbiota	Reducing inflammaging and enhancing gut integrity	[17]
Mouse	FMT from wild-type into progeroid mice	Delayed aging phenotypes and extended lifespan/restoration of secondary bile acid synthesis	Remodeling age-associated gut dysbiosis	[19]
Mouse	FMT from young into aged mice	Improved gut barrier function and reduced intestinal inflammation/restoration of mesenteric lymph nodes associated immunity and gut homeostasis	Reducing inflammaging and enhancing gut integrity	[21]
*C. elegans*	*E. coli* mutants producing excess colanic acid (CA)	Extended longevity/promoted mitochondrial dynamics and activated unfolded protein response by CA	Rescuing mitochondrial dysfunction and restoring proteostasis	[24]
*C. elegans*	Nitric oxide-producing Bacilli	Extended longevity/promoted thermotolerance via HSF-1 and DAF-16 transcription factors	Rescuing decreased stress resistance	[25]
*C. elegans*	*Lactiplantibacillus plantarum* producing 3-phenyllactic acid (PLA)	Extended healthspan/enhanced energy metabolism and stress resilience by PLA via stress-related transcription factors SKN-1/NRF2	Rescuing decreased stress resistance	[26]
*D. melanogaster*	Reduced *Acetobacter* via early-life oxidants exposure	Extended lifespan/ameliorate gut dysfunction	Rescuing immune deficiency	[30]

## Maternal factors affect early-life microbiome

**Fig. 1:**
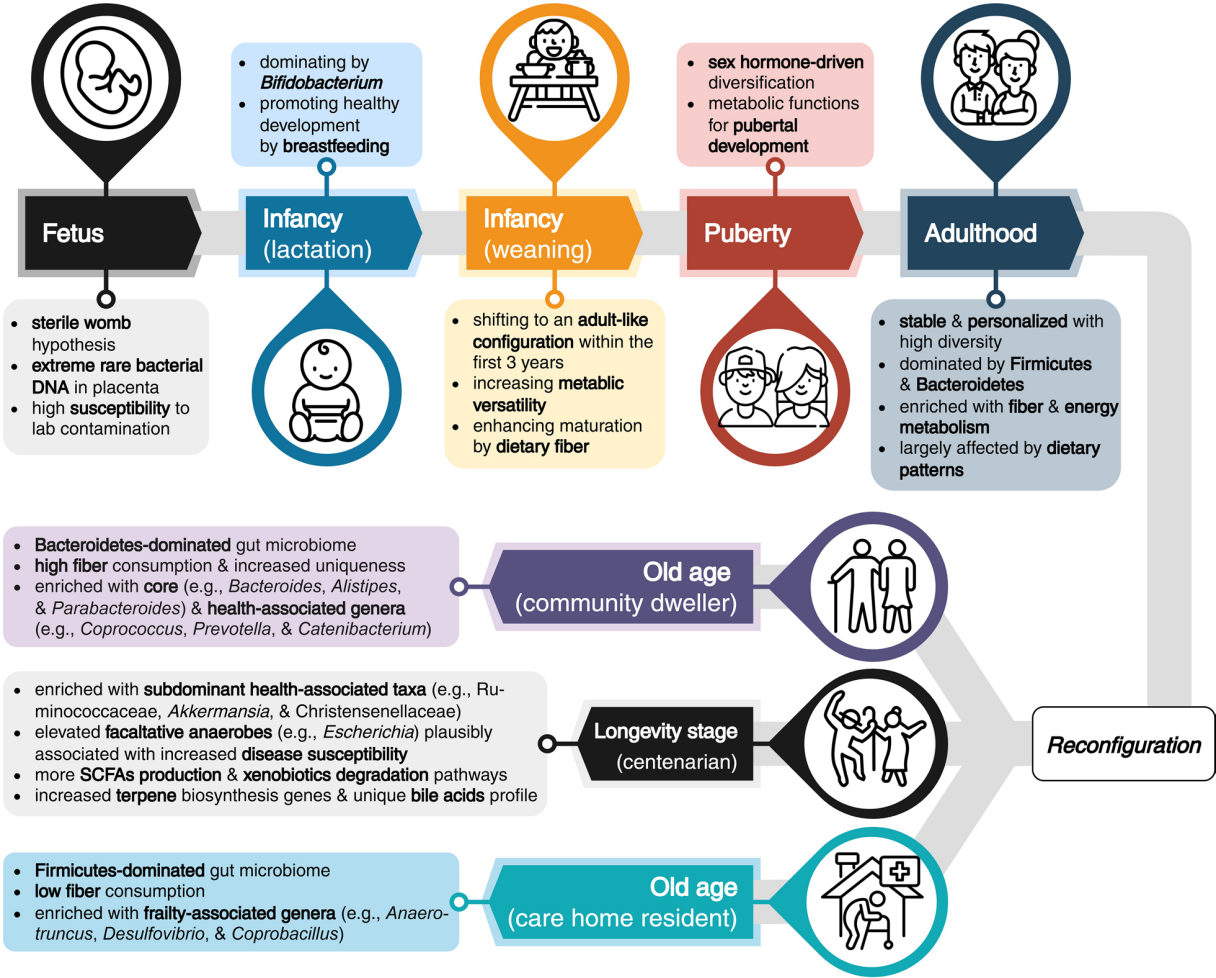
The human gut microbiome characteristics at different life stages. The characteristics are presented with key terms in bold typeface, following the order of compositional features, factors for modulation, and enriched metabolic function or metabolite profiles. Reconfiguration denotes the transitional stage involving the substitution of butyrate-producing taxa, where its success steers the elderly gut microbiome towards healthy aging, or vice versa, towards unhealthy aging. The icons were sourced from Flaticon.com, with credit to the following icon artists: Metami septiana, dwiangga.icon, Freepik, Eucalyp, kliwir art, mia elysia, and anilofex

While research has described the importance of the gut microbiome to health and its potential role in mitigating aging, in humans, the sources of an individual’s microbiome remain uncertain and debated within the literature. Over the past two decades, microbial presence has been reported in the prenatal intrauterine environment, including the placenta [34, 35], amniotic fluid [36], umbilical cord blood [37], and the fetus [38], challenging the widely accepted sterile womb hypothesis [39]. A later analysis of 537 placental biopsies found bacterial DNA to be extremely rare, with most instances of detection being attributed to contamination during delivery and laboratory processing, although *Streptococcus agalactiae* was detected as a non-contaminant in some cases but without significant association to pre-eclampsia or preterm birth [40]. Due to the inherently low bacterial biomass of prenatal samples, they are highly susceptible to contamination during collection and laboratory processing [41, 42] (Fig. 1). To avoid false-positive results, microbiome studies assessing low-biomass samples are recommended to be designed specifically to control for contamination and to adopt quantitative PCR with low detection limits and microscopic inspection before high-throughput sequencing [42]. Taken together, while sporadic bacterial DNA signals have been detected, the sterile womb hypothesis still largely holds. Despite some non-contaminant microbial signals have been identified, the placenta appears to act as a protective barrier for the fetus and may represent the furthest point bacteria can reach in the womb.

Although the question of when and through which interactions fetal microbiomes are acquired remains controversial, there is a general agreement that infants acquire their microbiome once they leave the womb. Delivery mode has been proven to affect the microbiome babies acquire, with vaginal delivery leading to an enrichment of vaginal species like *Lactobacillus* and *Prevotella*, and Caesarean section (C-section) favoring skin-associated species like *Staphylococcus*, *Corynebacterium*, and *Propionibacterium* [43]. As C-section infants were found to have an increased susceptibility to allergic diseases [44], the distinction in their initial microbiome has plausible long-term health effects. This is further supported by evidence showing that the early-life microbiome shapes gut mucosal immunity by modulating the accumulation and function of invariant natural killer T cells [45]. A subsequent study identified that a transient gut microbial dysbiosis within the first 100 days of life increased the risk of asthma, with the allergy-prone infant gut microbiome characterized by reduced level of *Lachnospira*, *Veillonella*, *Faecalibacterium*, and *Rothia* [46]. The gut microbiome of C-section and vaginal delivered babies were monitored and largely aligned after time, with differences observed between the groups at 1 week and one month after birth diminishing after one year. However, increased incidence of asthma was only found in those individuals that retained the C-section-associated microbiome at one year of age [47], reinforcing the relationship between the gut microbiome and health outcomes. Recently, the first-year gut microbiome has been proposed as a predictor of pediatric allergic disease by age five [48]. Therefore, appropriate maturation of the gut microbiome towards vaginal birth assemblages mitigates the increased asthma risk associated with C-sections, making the first year a critical period for nurturing the gut microbiome.

Human milk is another potential source of vertical microbial transmission, making it plausible source of or contributor to an infant’s gut microbiome. Lactic acid bacteria has been isolated from human milk [49], overturning its traditionally believed assumption of sterility [50], with an estimated 8 × 10^4^ to 8 × 10^6^ bacteria ingested daily in breastfed infants [51]. At the same time, lactic acid bacteria in human milk has been found to inhibit the growth of pathogenic *Staphylococcus aureus* [51], suggesting protective benefits for breastfed infants. While the microbiome of human milk is very diverse, it appears to be dominated by *Staphylococcus*, *Streptococcus*, *Bifidobacterium*, and *Lactobacillus* bacterial genera [52]. There is variation among the human milk microbiome of different mothers; however, these unique assemblages were found to be stable in each mother over the sampling period of 4 weeks [53]. Over longer periods, studies have found that breast milk microbiomes change throughout entire lactation periods, with stratification based on the mother’s weight, mode of delivery [54], and breastfeeding practices [55]. Notably, indirect breastfeeding (defined as at least one feeding of pumped milk in the preceding 2 weeks of sampling) was associated with the enrichment of potential pathogens and a depletion of the assumed positive *Bifidobacterium* [55]. In breast milk, the human milk oligosaccharides (HMOs) were recognized as prebiotics that promote the growth of beneficial bacteria [56], providing a potential modality for the observed higher abundance of *Bifidobacterium* and less *Clostridium* in the breastfed than formula-fed infants [57]. The benefits of *Bifidobacterium* to infants have been found to correlate with their ability to utilize HMOs and produce key metabolites like indole-3-lactic acid, leading to a healthier immune imprinting by reducing intestinal inflammation and thus lowering the risk of immune-related diseases later in life [58]. By clustering and trajectory modeling of the gut microbiome from nearly 1000 infants, breastfeeding, vaginal birth without antibiotic exposure, and having siblings were found to contribute to the most common trajectory associated with healthy development [59].

While differences have been observed in microbiomes related to delivery mode and milk source, the introduction of solid foods during weaning provides further influence on an infant’s gut microbiome. Research has shown that the cessation of breast milk coincides with the maturation of the infant gut microbiome [60, 61], transitioning from an infant-like to an adult-like composition within the first three years of life [62]. This maturation is accompanied by the growth of bacteria with greater metabolic versatility, facilitating carbohydrate utilization, vitamin biosynthesis, and xenobiotic degradation [63]. During this transition, the previously *Bifidobacterium*-dominated gut microbiome shifts toward a more taxonomically diverse community, enriched with Bacteroidaceae, Lachnospiraceae, and Ruminococcaceae, and shows an increased microbial alpha diversity [60], which likely underlies the expanded functional capacity. In paired, longitudinal monitoring of maternal and infant microbiomes, the median first appearance of shared Bacteroidales, Oscillospiraceae, and Lachnospiraceae strains was significantly earlier in mothers than in their infants, indicating that infants acquired these strains postnatally through cohabitation rather than vertically transferred at birth or through breastfeeding [64]. The timing of weaning was also recently reported to influence the gut microbiome, and early complementary feeding was associated with a higher BMI at the age of five [65]. Dietary fiber incorporation during weaning supported the gradual microbiome maturation in the infant gut [66], suggesting that the weaning practice may have lasting health implications for infants.

## Puberty comes with gut microbiome transition

Puberty represents a transitional stage from childhood to adulthood, during which hormonal fluctuations, growth spurts, sexual maturation, and metabolic reprogramming occur simultaneously. There is evidence supporting each of these physiological changes as impacting the gut microbiome. In contrast to the earlier-assumed concept that an adult-like gut microbiome is established after the first three years of life and remains stable thereafter, the gut microbiomes of adolescent children exhibit some differences compared to those of adults. In addition to *Ruminococcus*, *Faecalibacterium*, and *Roseburia* being the core taxa shared with adults, *Bifidobacterium* and *Clostridium* were found also abundant in adolescents [67]. Furthermore, the adolescent gut microbiome showed higher Shannon diversity and functional enrichments that support pubertal development (e.g., synthesis of vitamin B12 and folate) compared to that of adults [68]. Given that sex hormones are critical drivers of sexual dimorphism during puberty, investigations into their effects on the gut microbiome have not surprisingly revealed that they are reciprocally influential. The highly similar gut microbiome in male and female mice at weaning showed gender-dependent diversification at puberty [69], which was eliminated by castration [70]. Transplanting the gut microbiome from adult male mice to immature female mice elevated blood testosterone levels in recipients and conferred protection against type 1 diabetes [69]. The androgen-driven gut microbiome was thus plausibly protective, with bacteria lineages contributing to macrophage imprinting [70], consequently playing a partial role in the gender bias observed in autoimmune diseases.

With the ability to metabolize sex hormones, the gut microbiome appears to influence the timing and progression of puberty. The gut microbiome has been shown to be indispensable for sexual maturation in mice, as germ-free males demonstrated defective spermatid differentiation and females showed disorganized follicle structures, compared to their conventionally raised counterparts [71]. In humans, although found only in girls, the relative abundance of estrogen-degrading Clostridia increased and Bacteroidia decreased with pubertal development [72]. Among Clostridia, members belonging to the Ruminococcaceae family are suspected to affect pubertal timing by regulating sex hormone levels in the host. The gut microbiome has also been reportedly associated with pubertal obesity [73] and behavioral changes [74], suggesting that microbiome manipulation during puberty could promote healthy adulthood maturation [75].

## The gut microbiome in adulthood is stable and personalized

The adult gut microbiome has been investigated through several population-based cohorts worldwide, including American [76–78], European [79, 80], Chinese [81–83], British [84], Belgian [85], Dutch [86, 87], and Japanese [88, 89] adults. From these studies the adult gut microbiome was estimated to harbor 1000–1150 prevalent bacterial species collectively and at least 160 species per individual [79], dominated by Firmicutes and Bacteroidetes phyla, followed by Actinobacteria, Proteobacteria, and Verrucomicrobia [90]. At the genus level, *Bacteroides*, *Faecalibacterium*, *Blautia*, *Roseburia*, and *Coprococcus* emerged as the core genera defined by 95% prevalence with known taxonomy [85]. On a global scale, *Bacteroides*, *Bifidobacterium*, *Shigella*, *Faecalibacterium*, and *Prevotella* were recently identified as the top five abundant genera in the human gut microbiome [91]. Despite this, the relatively low number of unique genera compared to species in the human gut microbiome suggested that the underlying diversity was concentrated at the species and strain level [92].

The gut microbiome in healthy adults is more stable than that in early life [93], as evidenced by the greater interpersonal variation among infants compared to adults [62]. Consistent with the ecological theory that diversity is positively linked to ecosystem stability [94], the richness and diversity of the adult gut microbiome were found to gradually increase after infancy, plateau at adolescence, and remain stable throughout the lifetime [95]. One study extrapolated this by suggesting that most gut strains persist as residents for decades in the same individual [96]. Despite this plateau associated with the physiological development towards adulthood, the gut microbiomes in healthy individuals still remain diverse, and it is this diversity that confers microbial resilience, helping the host to withstand and recover from perturbations while maintaining its functionality and adaptability [97]. The adult gut microbiome also appears to be more robust compared to that of infants, showing a quicker recovery after a single antibiotic exposure [98], although the effects of antibiotic treatment lasted longer in adults than in infants [99] and depended on the type of antibiotic used [100]. Given the taxa-dependent ability for persistent colonization [101], the same perturbation can have differential impacts on gut strains. However, even in the absence of perturbation, age alone is sufficient to cause fluctuations in the gut microbiome [102, 103], and the smaller intra-individual variation compared to inter-individual variation indicates a highly personalized nature of the gut microbiome.

The variation in gut microbiomes observed among adults may have a genetic link. Population-scale genome-wide association studies (Table 2) have demonstrated that monozygotic twins exhibit more similar gut microbial compositions than dizygotic twins, indicating a genetic component in the heritability of gut microbiome, with Christensenellaceae identified as the most heritable taxon [104]. Specific host genetic variants also affect the microbial abundance. For example, polymorphisms in the lactase (LCT) gene, which determine lactase persistence, were associated with *Bifidobacterium* levels in lactose-consuming individuals [105–107]. Additionally, *Bacteroides* species have been shown to have different abundances related to ABO blood group genotypes [108–110]. Further, a set of 42 single-nucleotide polymorphisms (SNPs) collectively explained over 10% of gut microbial β-diversity [111]. When considering more genetic variants, host genetic effects additively accounted for up to 20% of the variation in such diversity [112], and 11% of the variance between *Bacteroides*- and *Prevotella*-dominated microbiomes was also attributable to the top two genetic loci linked to host enterotypes. Despite the influence of these genetic associations, non-genetic factors related to diet and lifestyle have been demonstrated to exert an equal or greater influence [113], accounting for more than 20% of the variance in the gut microbiome, while the average heritability of the microbial taxa is estimated to be only 1.9% (in TwinsUK data).

**Table 2:** Representative gene–environment (GxE) studies illustrating interactions between host genetic factors and gut microbiome variation

Study/cohort	Population	Host genetics factor	Microbial trait affected	Key findings	References
TwinsUK	British adults	Twin zygosity	Taxon heritability	Monozygotic twins share more similar gut microbiota than dizygotic twins, and Christensenellaceae is the most heritable taxon	[104]
HMP, TwinsUK, and LifeLines-DEEP	American, British, and Dutch adults	SNPs in lactase (LCT) gene locus	*Bifidobacterium* abundance	LCT polymorphisms (lactase persistence) strongly associate with *Bifidobacterium* levels in lactose-consuming adults	[105–107]
PopGen, LifeLines-DEEP, and FINRISK	German, Dutch, and Finnish adults	ABO genotypes	*Bacteroides* spp. abundance	*Bacteroides* species are enriched or depleted in individuals with different ABO genotypes	[108–110]
PopGen and FoCus	German adults	SNPs	Beta diversity	A set of 42 human SNPs collectively accounts for over 10% of the variation in β diversity	[111]
4D-SZ	Chinese adults	Genetic variants (including common variants, rare variants, and copy number variation)	Beta diversity and enterotypes	Genetic variants additively contribute 20% of the variation in β diversity, and the top two loci associated with enterotypes explain 11% of the variance in the *Bacteroides* versus *Prevotella* enterotype dichotomy	[112]
Israeli cohort	Israeli adults	SNPs	Beta diversity and taxon heritability	Non-genetic factors contribute over 20% of inter-person microbiome variation, and the average heritability of gut microbial taxa is only 1.9% (in TwinsUK data)	[113]

With respect to metabolic functions, the pathways with differential enrichments reflect developmental priorities across different life stages. In comparison to infants, the adult gut microbiome preferred different genes involved in vitamin, amino acid, and carbohydrate metabolisms [62]. Specifically, genes in vitamin B9 (folate) biosynthesis, cysteine metabolism, and lactic acid bacterial fermentation were more abundant in infants. In contrast, genes associated with vitamin B12 (cobalamin), B7 (biotin), and B1 (thiamine) biosynthesis, as well as arginine, glutamate, aspartate, and lysine metabolism, and methanogenesis were enriched in the adult gut microbiome. By correlating 104 bacterial species with age-dependent abundance with metabolic functions, the adult gut species were found to have more genes related to fiber metabolism than infants [95], correlating with the fiber content in diets. In addition, a greater abundance of pathway counts in energy metabolism and fewer in carbohydrate metabolism were among the top functional categories distinguishing the gut microbiomes of adults from those of infants [114]. These differences were also attributable to their dietary patterns, which in turn influenced the gut microbiome composition.

## The elderly and centenarian gut microbiomes exhibit both differences and similarities

The elderly age-class is conventionally defined as individuals aged 65 years or older, a period where individuals generally switch from working to retirement, experience a change in dietary patterns, undergo physiological decline, and increase medication use. These changes individually and in concert are expected to alter the gut microbiomes, making the elderly cohort not simply an age class but a biologically unique group.

An early study compared the gut microbiomes in Italian adults, elderly, and centenarians, finding that the elderly had more *Clostridium leptum* and *Akkermansia* than adults, and that centenarians had an increase in facultative anaerobes, primarily Proteobacteria, compared to the other two groups [115]. Shotgun metagenomic sequencing on a subset of the same cohort found *Escherichia* and *Ruminococcus* to be more abundant in the centenarian gut microbiome, whereas *Faecalibacterium*, *Eubacterium*, and *Bifidobacterium* were more abundant in the elderly [116]. The increased abundance of *Escherichia*, a pro-inflammatory proteobacterium, in centenarians draws attention to its potential role in aging. Among species in this genus, *Escherichia coli* is the most prevalent and well-studied, and although typically a harmless gut commensal, some *E. coli* strains have the potential to become pathogenic under susceptible host conditions, acting as pathobionts. Polyketide synthase (*pks*)-positive *E. coli*, which produces the genotoxin colibactin, have been shown to enhance sarcoma invasiveness in interleukin-10-deficient mice [117]. A later study in colorectal cancer mouse models found that *pks*^+^ *E. coli* promoted tumor growth by inducing cellular senescence [118]. Invasive *E. coli* can also contribute to tumorigenesis by suppressing epithelial autophagy and promoting oxidative stress in chemically induced cancer models [119]. Moreover, colonization with a wild-type microbiome led to elevated *Escherichia* abundance and exacerbated colitis in transgenic mice with impaired gut barrier function [120]. Therefore, the increase of *Escherichia* and pro-inflammatory microbes in centenarians may not only reflect a unique, balanced gut ecosystem supported by higher immune tolerance in long-lived individuals but also imply an elevated susceptibility to pathological conditions if gut homeostasis is disrupted.

In an Irish cohort, the generic microbiome feature was approached based on sample prevalence. By focusing on taxa present in over 50% of individuals, *Bacteroides*, *Alistipes*, and *Parabacteroides* were found to be the core microbiome, together contributing 53% of the abundance in the elderly but only 8–27% in younger adults on average [121]. The same team subsequently discovered that the elderly had gut microbiome differences associated with their dietary pattern, which was initially explained by residence locations of individuals in the study [122]. Community-dwelling elders, who had better health parameters than care home residents, harbored a microbiome configuration dominated by phylum Bacteroidetes and enriched with genera *Coprococcus* and *Roseburia* which was similar to younger adults and indicative of healthy aging. On the other hand, the gut microbiome of care home residents, dominated by phylum Firmicutes and enriched with genera *Parabacteroides*, *Eubacterium*, *Anaerotruncus*, *Lactonifactor*, and *Coprobacillus*, differed from the adult cohort and was associated with frailty. Further investigation suggested that a high-fiber diet was a credible and rational determinant of the gut microbiome for healthy aging, as community dwellers consumed more fiber than care home residents [122]. Cluster analysis identified four modules associated with aging in the elderly gut microbiome [123], among which the core (*Bacteroides*, *Alistipes*, *Parabacteroides*, *Faecalibacterium*, and *Ruminococcus*) and diversity-associated modules (*Coprococcus*, *Prevotella*, and *Catenibacterium*) were linked to health and healthy food diversity, while the long-stay-associated module (*Anaerotruncus*, *Desulfovibrio*, and *Coprobacillus*) showed a positive correlation with frailty.

Centenarians form a unique group among the elderly due to their exceptional longevity and often healthier aging profiles, such as a lower incidence of chronic illness [124], thus providing a distinct viewpoint on factors associated with longer lifespans and healthy aging. An Italian study found that centenarians had a distinct gut microbiome configuration compared to that of adults and elderly, characterized by a slight reduction in the predominant families Bacteroidaceae and Lachnospiraceae and increases in the subdominant taxa [125], among which the health-associated members, such as *Akkermansia*, *Bifidobacterium*, and Christensenellaceae, appeared to increase gradually from centenarians to semi-supercentenarians (aged 105–110 years). Concordantly, when compared to younger elders and adults, Chinese long-lived individuals (aged 90 years or older) shared several gut microbial features with Italian centenarians, including significant increases in health-associated taxa (e.g., *Clostridium* cluster XIVa, Ruminococcaceae, A*kkermansia*, and Christensenellaceae) and microbial richness [126]. The increase of Ruminococcaceae in Chinese centenarians was associated with a high-fiber diet, suggesting the potential of fiber consumption to modulate the centenarian gut microbiome for healthy aging [127]. Among Japanese individuals, increases in *Akkermansia*, Christensenellaceae, Ruminococcaceae, and *Clostridium*, along with decreases in Bacteroidaceae and Lachnospiraceae, were also reported among nonagenarians and centenarians compared to younger elders [128], while *Bifidobacterium* diminished after the age of 90. Beyond taxonomy composition, centenarians were generally recognized to have a gut microbiome with elevated richness and diversity [129].

The gut microbiome markers of healthy aging have recently been extensively studied using large population cohorts. Among Chinese individuals, the centenarian gut microbiome exhibited features similar to those of young adults (20–44 years) compared to old adults (66–85 years), manifested by elevated species evenness, more prevalent *Bacteroides*-dominated enterotypes, enriched beneficial *Bacteroides* species, and reduced opportunistic pathogens [130]. Meanwhile, a 1.5-year follow-up among a subset of these Chinese centenarians reported an enhanced evenness, a stable *Bacteroides* abundance, and reduced intraindividual variation, suggesting potential microbiome indicators for healthy aging. Based on three US-based population cohorts, the degree of uniqueness—defined as the minimum Bray–Curtis distance between an individual’s gut microbiome and all others in the cohort—was found to increase with age [131] and was predictive of better survival outcomes among healthy elderly individuals aged over 85. The association between age and microbiome uniqueness was attributed to the reduced core *Bacteroides* species. In contrast, individuals in poorer health demonstrated declines in other core taxa, such as the genera *Lachnoclostridium* and *UBA1819* (within the family Ruminococcaceae). A later thorough investigation analyzing over 20,000 fecal metagenomes identified that different uniqueness measures (i.e., Bray–Curtis, Jaccard, Aitchison, and Kendall dissimilarity) positively correlated with age, but primarily in European and North American individuals [132]. Among all tested measures, only Kendall uniqueness was proposed as indicative of unhealthy aging, rather than healthy aging, reflected by the reduction of the core microbiome, loss of diversity, and increase in disease-associated taxa. Overall, these findings indicate that choices of uniqueness metrics meaningfully affect interpretations of microbiome aging patterns, and that population-level variation may also impact their interpretability.

Plasma samples collected in the Framingham Offspring Study were analyzed to identify metabolic markers associated with longevity, finding that lower concentrations of circulating isocitrate and taurocholate were associated with higher odds of living to age 80 [133]. Fecal metagenomic analysis revealed a higher metabolic diversity, more glycolysis and short-chain fatty acid (SCFA) production genes, but lower fiber and galactose degrading enzymes in Sardinian centenarians compared to the elderly and younger adults [134]. Moreover, an enrichment of xenobiotics degradation and metabolism pathways, oxidoreductases, and multiple health-promoting species were found in Chinese centenarians [135], likely due to their longer history of xenobiotics exposure than those in the adult age groups. Japanese centenarians had a unique gut microbiome reportedly capable of generating various isomers of lithocholic acid (LCA), which decrease the risk of pathogenic infection [136], implying that intestinal homeostasis maintained by secondary bile acids contributes to the health of centenarians. By integrating six cohorts from Japan, Italy, and China, biosynthetic gene clusters for terpene and type I polyketide synthases were consistently found to be enriched in nonagenarians and centenarians [137]. Terpene biosynthesis supports healthy aging largely through its role in producing tetraterpenoids, the precursors of vitamin A and antioxidants, thereby mitigating age-related cellular and systemic damage.

Studies have demonstrated that elderly enterotypes were predictable by their historical variables [138], suggesting that long-term lifestyle practices cooperatively shape the current gut microbiome. Thus, the centenarian gut microbiome may reflect their lifelong healthy lifestyles, with its unique features not only serving as important biomarkers of healthy aging but also helping to understand the biological mechanisms for longevity.

## The gut microbiome mechanistically links to the aging process

**Fig. 2:**
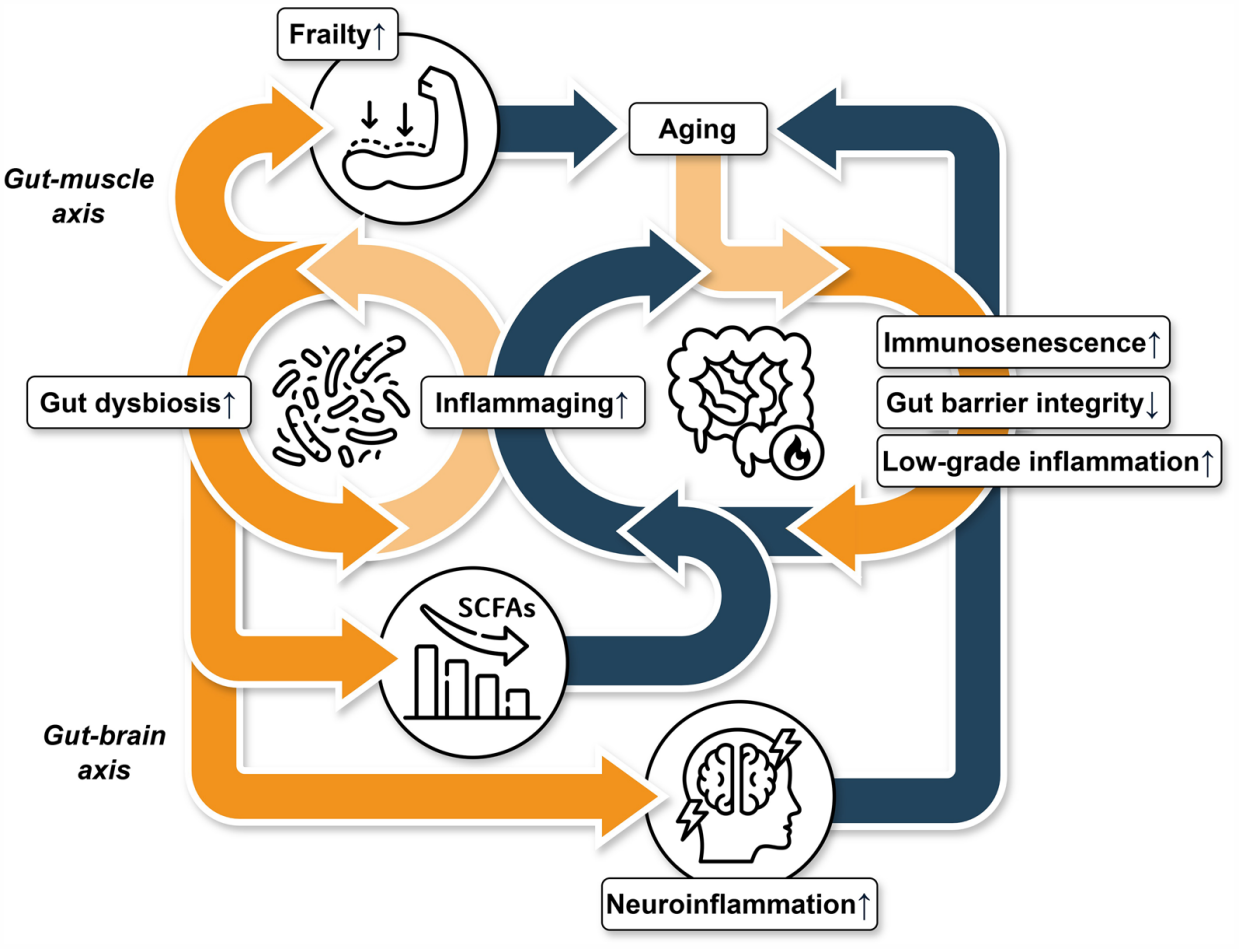
Mechanistical contributions of the gut microbiome to the aging process. As people age, immunosenescence occurs with a concomitant decline in gut barrier integrity and an onset of low-grade inflammation, leading to inflammaging, which contributes to gut dysbiosis then a decrease in SCFA production, further exacerbating inflammaging. The dysbiotic gut microbiome accelerates the aging process, contributing to muscle loss via the gut-muscle axis, leading to frailty, and inducing neuroinflammation through the gut-brain axis, resulting in cognitive decline. The icons were sourced from Flaticon.com, with credit to the following icon artists: Culmbio, C-mo Box, Freepik, Hat Tech, and Dewi Sari

The gut microbiome is recognized as a critical modulator of aging processes and evidence has suggested that age-related low-grade inflammation, termed ‘inflammaging’, is driven in part by the alterations in the gut microbiome [15, 139]. One example is the significant changes observed in centenarians, especially the decrease in beneficial Firmicutes and the increase in pro-inflammatory Proteobacteria [115]. Inflammaging worsens gut barrier integrity, resulting in gut dysbiosis that further exacerbates inflammation, creating positive feedback of health degradation with age [18]. Meanwhile, this process is closely associated with immunosenescence, the age-related decline of immune function that shows impaired immune responses to infections, thereby elevating susceptibility to inflammation [140]. These conditions are not independent during the process of aging but rather intertwined, perpetuating and exacerbating gut dysbiosis with age [141] (Fig. 2). The most dominant impact of the age-associated microbiome changes is the decrease in the production capability of SCFAs, which play an essential role in maintaining intestinal homeostasis [142]. In infants, the dominant *Bifidobacterium* species produce lactate and acetate from milk oligosaccharides. After weaning, the diversified gut microbiome gradually becomes dominated by Firmicutes, which includes various species capable of degrading complex fibers, resulting in the production of butyrate and other SCFAs [143]. The elderly gut microbiome shows a further compositional reconfiguration (Fig. 1), wherein adult-predominant butyrate-producing taxa (e.g., *Coprococcus*, *Faecalibacterium*, *Roseburia*, and *Eubacterium*) are replaced by subdominant taxa (e.g., *Odoribacter*, *Oscillospira*, *Butyrivibrio*, and *Butyricimonas*) [14]. This reconfiguration, a compositional shift involving the replacement of dominant adult-associated SCFA producers with less prevalent taxa of similar function, appears to be the turning point for healthy and unhealthy aging. Since SCFAs have anti-inflammatory effects on the immune system [144], elderly individuals in whom this transition does not sufficiently preserve SCFA production may experience intensified inflammaging due to heightened immune sensitivity.

The gut-brain axis is an important pathway by which the gut microbiome mediates aging since its metabolites, including SCFAs, neurotransmitters, and their precursors, are involved in regulating brain functions and cognition [145]. As a result of inflammaging, disruption of the intestinal barrier and gut dysbiosis increase pro-inflammatory components and metabolites that enter circulation, eliciting neuroinflammation that eventually leads to brain aging associated with neurodegenerative diseases [146]. For example, microbial metabolites derived from dietary tryptophan have been shown to act as ligands that trigger gene expression in microglia cells, reducing the central nervous system inflammation [147]. In parallel, SCFAs released by the gut microbiome control microglial maturation and function [148]. Among these SCFAs, acetate was later identified as the major molecule responsible for microglial homeostasis [149]. Additionally, propionate enhances immune cell balance, leading to improvements in immunological and neurodegenerative parameters in patients with multiple sclerosis [150]. Supplementation with a mix of acetate and propionate was found to restore cognitive and social behavioral deficits in mice induced by maternal obesity [151]. These studies suggest that gut dysbiosis may accelerate neuroinflammation by altering gut microbiome metabolism along the aging process.

While the neuroprotective and anti-inflammatory properties of SCFAs are widely acknowledged, recent evidence indicates that the effects exerted by SCFAs on brain health may be context-dependent and potentially detrimental. In Alzheimer’s disease (AD), SCFA supplementation increased gliosis and tau pathology in germ-free (GF) TE4 mice [152]. Similarly, SCFAs were shown to exacerbate amyloid-β plaque deposition in both GF and specific pathogen-free (SPF) APP/PS1 mice [153]. In Parkinson’s disease (PD), SCFAs have been linked to α-synuclein (αSyn) aggregation and motor deficits. Using GF Thy1-αSyn mice, one study demonstrated that SCFA supplementation recapitulated the colonized phenotypes, including microglial activation and neuroinflammation, ultimately leading to worsened motor performance [154]. Clinically, plasma SCFA levels have been reported to be elevated in PD patients compared to normal controls [155], and in particular plasma acetate, propionate, and valerate are positively correlated with disease severity as quantified by various PD rating scales [156]. Together, these findings suggest the double-edged nature of SCFAs in brain aging and disease (Table 3); SCFAs can promote neuroprotection under specific conditions, but they may also contribute to disease progression if neurodegenerative pathways are activated.

**Table 3:** Beneficial and detrimental roles of SCFAs in brain aging and disease

Aspect	Beneficial roles of SCFAs	Detrimental roles of SCFAs
Neuroinflammation and immunity	●Regulate microglial maturation and function (majorly acetate) [148, 149] ●Improve immune balance in patients with multiple sclerosis (propionate) [150]	●Induce neuroinflammation in PD models [154]
Cognitive and behavioral effects	●Restore cognitive and social behavior in offspring of obese mice (acetate and propionate) [151]	●Associate with worsened motor performance in PD models [154]
Neurodegenerative pathology	●Support microglial homeostasis (acetate) [149]	●Increase gliosis and tau pathology in AD models [152] ●Exacerbate amyloid-β plaque deposition in AD models [153] ●Promote α-synuclein aggregation in PD models [154]
Clinical correlation	●Provide potential benefits under balanced immune and microbial states	●Elevate in plasma of PD patients and correlate positively with disease severity [155, 156]

The physical decline and loss of body resilience during aging are linked to frailty, a clinical syndrome characterized by increased vulnerability to stressors and negative health outcomes. Frailty has been associated with age-related gut dysbiosis, exemplified by losing diversity and beneficial bacteria like *Faecalibacterium prausnitzii*, and acquiring disease-associated species like *Eubacterium dolichum* and *Eggerthella lenta* [157]. A meta-analysis similarly revealed more pathogenic and fewer commensal species in frail compared to non-frail older adults [158]. Despite these observations illuminating an association, the mechanisms connecting the gut microbiome to frailty are relatively unexplored.

It is known that the gut microbiome influences skeletal muscle mass and function [159], forming the gut-muscle axis, which is intricately connected through various microbial metabolites, such as SCFAs [160]. A reduced level of fecal butyrate has been associated with low muscle mass in elderly Taiwanese individuals [161] and a longitudinal analysis revealed positive associations between SCFA intake and muscle strength among community-dwelling older Japanese adults [162]. The beneficial effects of SCFAs have been also recently linked to the activation of mTOR signaling pathways, which improve mitochondrial biogenesis and protein synthesis of atrophic muscle [163]. Thus, gut dysbiosis induced by inflammaging contributes to frailty by impairing muscle functionality. Since there is still a lack of effective treatment for frailty, the gut microbiome may present a promising avenue for intervention, potentially promoting healthy aging by enhancing muscle functionality to mitigate frailty.

## The gut microbiome emerges as a modifiable therapeutic target for healthy aging

**Fig. 3:**
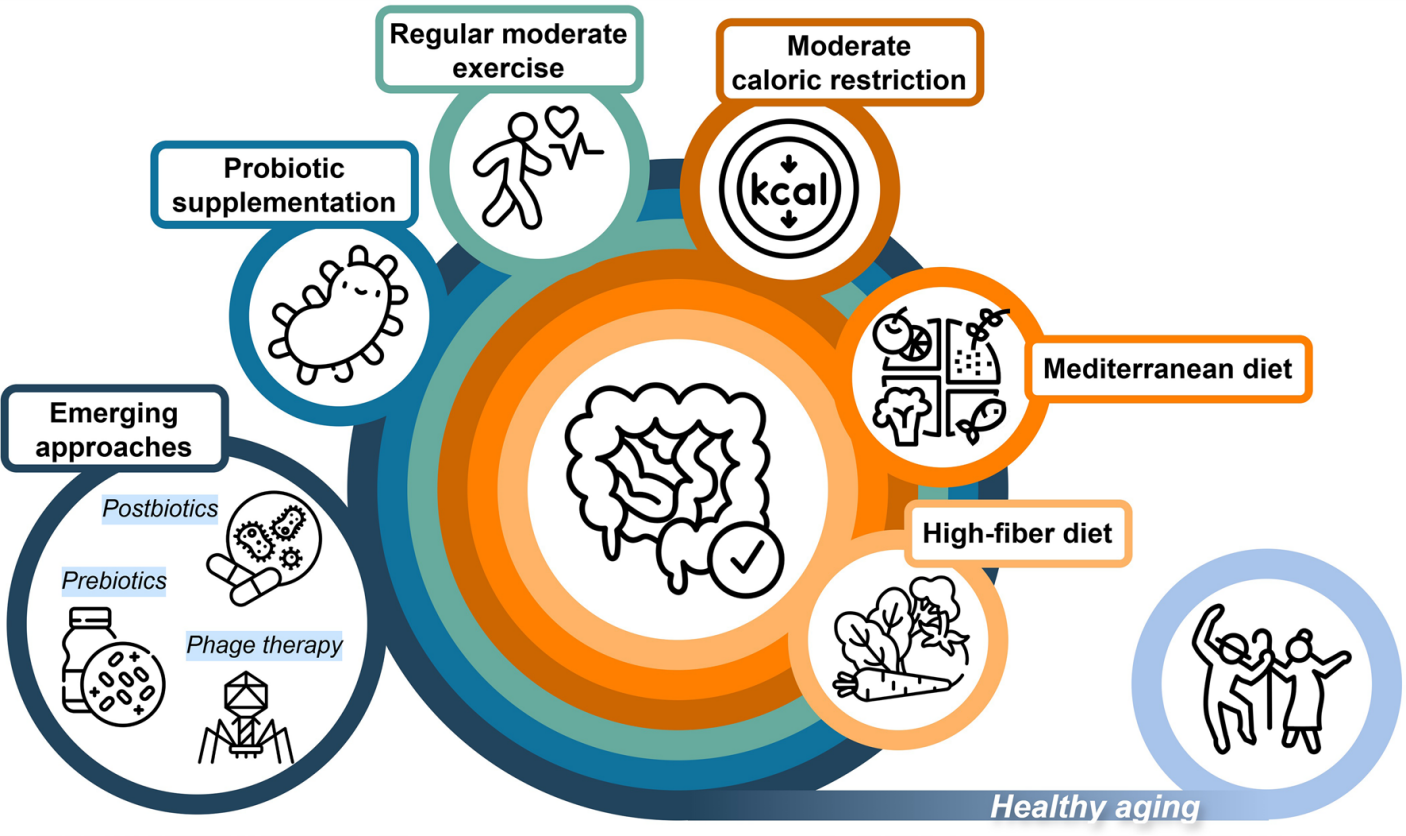
Potential interventions to modify the gut microbiome for healthy aging. The gut microbiome is largely modulated by dietary patterns, with high-fiber and Mediterranean diets showing benefits in promoting healthy aging. Moderate caloric restriction should be practiced cautiously due to the risk of muscle loss. Regular exercise with moderate intensity is also recommended. Probiotics supplementation offers beneficial effects that vary depending on heterogenous trial designs. Lastly, emerging approaches, including prebiotic and postbiotic supplementation and phage therapy, require further clinical trials to validate their safety and efficacy. The icons were sourced from Flaticon.com, with credit to the following icon artists: Culmbio, Icongeek26, Becris, Freepik, Iconjam, bsd, spacepixel, and anilofex

Collectively, studies investigating the association between the gut microbiome and human physiology underscore its indispensable role in healthy aging. The dynamic and responsive nature of these associations stand out as a prospective area for future interventions. Among accessible practices, dietary habits seem to be the most relevant driver dictating the gut microbiome [164, 165]. Specifically, a high-fiber diet has been associated with healthy aging in the elderly [122] (Fig. 3), consistent with the reported correlations between vegan-associated gut microbes and favorable cardiometabolic markers [166]. Building on the documented beneficial effects of the Mediterranean diet in shaping a health-promoting gut microbiome [167], this dietary pattern has been a focus of research for its purported impact on healthy aging. A one-year, large-scale clinical trial involving elderly participants across five European countries, whose diets were switched to a Mediterranean-style diet, demonstrated a remodeling of the gut microbiome toward a configuration enriched with beneficial taxa, which showed positive associations with reduced frailty and improved cognition [168]. Furthermore, indices evaluating global cognition and episodic memory also improved with increasing adherence to a Mediterranean diet [169], suggesting that a long-term practice of dietary intervention amplifies its effectiveness over time [170]. Supporting the long-term effects of diet, a thirty-year follow-up study on a US population confirmed that adhering to a healthy dietary pattern supports healthy aging, characterized by living to 70 years of age free of chronic diseases and maintaining cognitive, physical, and mental competence. These healthy dietary patterns emphasize a higher intake of fruits, vegetables, whole grains, unsaturated fats, nuts, legumes, and low-fat dairy products, while limiting trans fats, sodium, sugary beverages, and red and processed meats [171].

Dietary restriction has recently been found to significantly increase lifespan in mice, with a 40% caloric restriction resulting in the longest extension [172]. While much attention has been given to the relationship between caloric intake and body mass or obesity, there exists strong evidence to support that caloric restriction also imparts a positive influence on the gut microbiome. Interestingly, studies have found that different dietary restriction regimens (i.e., fasting and caloric restriction) induced similar modifications in the gut microbiome composition and metabolic potential in mice [173]. These microbiome changes were correlated with physical parameters and health phenotypes in aged mice but did not directly contribute to longevity, clearly suggesting that the benefits of microbial modulation on lifespan are mediated through improvements in physical conditions. Evidence in humans also supports the beneficial effects of caloric restriction on the gut microbiome, as demonstrated by a one-year intervention in obese adolescents that increased the abundance of butyrate-producing bacteria [174]. A three-month trial for the Mediterranean diet reduced body weight and fat mass, together with losing obesity-associated taxa in obese adults [175]. However, the effects of caloric restriction in the elderly remain to be explored. Although there are potential benefits, such as slow cellular aging, caloric restriction in the elderly also increases mortality from reduced body mass index and impaired muscle mass and strength. Other complementary strategies (e.g., resistance exercise and an increased protein intake) are also important during caloric restriction to balance its benefits and risks and optimize the health gains [176].

Regular exercise is widely known as being beneficial to human health. During exercise, the contraction of skeletal muscles releases myokines [177], which sustain the hallmarks of health by facilitating muscle-organ crosstalk [178], including interactions with the gut. Exercise-induced alterations in the gut microbiome have demonstrated positive effects on human health [179], and exercise has therefore been recommended as a therapeutic intervention for disease prevention [180]. In mouse models, exercise at different intensity levels enhanced the growth of beneficial gut bacteria [181] and exercise in combination with a shift to a healthy diet was found to effectively reshape the gut microbiome, providing metabolic benefits to recipient mice following microbiome transplantation [182].

In humans, studies looking at the gut microbiome of professional athletes revealed higher alpha diversity, lower inflammatory markers, a greater abundance of *Akkermansia* [183], and elevated fecal SCFA levels [184] compared to sedentary controls. Among non-athlete adults, although increased alpha diversity was not observed, *Akkermansia* was consistently responsive to moderate physical activity in premenopausal women [185] and endurance exercise in overweight women [186]. Recently, the gut microbiome from endurance athletes was found to improve insulin sensitivity when transplanted into mice [187]. In older adults, a six-month exercise intervention increased the abundance of gut health-associated taxa (e.g., *Bifidobacterium*, *Oscillospira*, and *Anaerostipes*) and raised fecal butyrate levels compared to their sedentary baseline [188]. Furthermore, daily or regular exercise in the elderly (over 60 years of age) was associated with a gut microbiome configuration similar to that of younger adults (aged 18 − 60 years old) and increased alpha diversity in overweight elderly individuals compared to those who rarely or never exercised [189]. Of note, the heat and ischemia induced by intense exercise can lead to the thinning of gut mucus [190] and increased microbial contact to the gut mucosal immune system [179], consistent with findings that prolonged, high-intensity exercise causes temporary gut dysfunction [191] and increased gut permeability and inflammation [192] in healthy adults. Although these detrimental effects are transient and generally benign, in the absence of evidence proving the health benefits of high-intensity exercise for the elderly, regular low- to medium-intensity exercise or endurance training is recommended as a safer approach to improving the gut microbiome and healthy aging.

Probiotic supplementation has arisen as an additional intervention option for healthy aging by modulating the gut microbiome. Several clinical trials have reported benefits in the elderly, but the overall evidence remains modest [193, 194]. Regarding age-related conditions, supplementing probiotics demonstrated improved gastrointestinal symptoms from Parkinson’s disease [195], enhanced muscle mass and function in participants with sarcopenia [196], reduced infection in care home residents, and accelerated recovery from fractures [197], but limited improvements in inflammation markers [198] and cognition [199]. For clinical trials targeting immune function, prebiotics and/or probiotic supplementation demonstrated high safety in the elderly, except in severely ill patients with pancreatitis, and two-thirds of these trials reported improved immune function in participants [141]. If effective probiotic supplementation is defined as successful colonization (or retention) in the gut, it depends highly on the host and gut microbiome features; only 60% of healthy individuals were permissive to probiotics during supplementation, and there was no significant difference from the placebo group after supplementation ceased [200]. Recently, a cohousing study in mice found that the elderly gut microbiomes were less modifiable compared to young gut microbiomes, with young mice acquiring gut microbiomes similar to that of the elderly group, but not vice versa [173]. Although the plasticity of the elderly gut microbiome remains unclear, it is likely to be more resistant to adopting new microbes than that of younger adults. Therefore, probiotic-based interventions in the elderly are expected to have limited efficacy in achieving colonization unless accompanied by prolonged supplementation, which may mimic the effects of consistent dietary practices.

Beyond these conventional approaches, the use of prebiotics (i.e., food for probiotics) and postbiotics (i.e., beneficial microbial-derived metabolites or inactivated microbes [201]) is gaining recognition for their potential to modulate the gut microbiome and promote healthy aging. In aged mice, supplementation with the prebiotic galacto-oligosaccharide restored gut homeostasis by strengthening intestinal epithelial integrity and promoting mucus production [202]. Resistant starches derived from beans and pulses positively modulated the gut microbiome composition, reinforced intestinal epithelial barrier integrity, and attenuated inflammation in the aging mouse model [203], and in particular lentils and chickpeas showed more favorable outcomes in postprandial glycemic control. Incorporating the soluble fiber inulin into a high-fat diet also improved gut and metabolic health in aged mice humanized with the pooled microbiome from healthy older adults, as indicated by increases in beneficial bacterial taxa and metabolites linked to a healthy metabolic state [204]. Furthermore, a clinical trial in elderly women demonstrated that consuming a synbiotic drink (probiotics + dietary fibers) resulted in improvements in fasting blood glucose, creatinine, and alkaline phosphatase levels during the intervention period [205], indicative of enhanced glycemic control, kidney function, and systemic metabolic health.

Studies exploring the efficacy of postbiotics have identified a range of mechanisms through which these compounds promote healthy aging. In elderly mice, heat-killed *Lactobacillus paracasei* D3-5 was shown to improve gut health, as well as physical and cognitive functions. Further investigation revealed that lipoteichoic acid, a cell wall component of the bacterium, was the key effector sufficient to reduce gut inflammation via NF-κB inhibition and enhance gut barrier integrity by promoting mucin production through the TLR2–p38 MAPK signaling pathway [206]. Commensal-derived indole-3-carboxaldehyde was shown to support gut homeostasis by promoting intestinal cell turnover and goblet cell differentiation via the aryl hydrocarbon receptor and IL-10 axis [207], which are critical for maintaining the gut barrier and immune balance in an aging intestine. Similarly, urolithin B, another microbial metabolite, was found to reduce inflammation by enhancing anti-oxidation capability and improve barrier function through the HMGB1–TLR4–NF-κB pathway in aging mice [208]. More recently, the heat-killed *Bifidobacterium longum* and *Lactobacillus acidophilus*, when combined with exercise, attenuated NF-kB-associated inflammation and improved mitochondrial homeostasis and Alzheimer’s pathology in the transgenic AD mouse model [209]. Together, these preclinical studies provide a strong foundation describing prebiotics and postbiotics as promising microbiome-targeted interventions to support healthy aging by restoring gut homeostasis, strengthening barrier function, reducing inflammation, and lowering age-associated neurodegeneration. Unlike live probiotics, these represent potentially safer, more stable, and mechanism-driven strategies.

Finally, bacteriophage (or phage) therapy—using viruses that infect bacteria to treat infections—is gaining renewed attention due to the growing crisis of antibiotic resistance and is emerging as another microbiome-based therapeutic approach [210, 211]. The high specificity of phages to their bacterial host allows them to be used to target and eliminate specific bacteria without disrupting the entire microbiome. Leveraging this host specificity, phages targeting cytolytic *Enterococcus faecalis* decreased liver cytolysin and abolished ethanol-induced liver disease in humanized mice colonized with the microbiome from patients with alcoholic hepatitis [212]. Other preclinical studies have shown that phage therapy effectively attenuates disease severity and suppresses corresponding *Klebsiella pneumoniae* strains associated with inflammatory bowel disease [213], primary sclerosing cholangitis [214], and non-alcoholic fatty liver disease [215], while also demonstrating safety in a human trial [213]. However, the limited efficacy of phage therapy targeting *E. coli* has been reported in clinical trials in individuals with self-reported gastrointestinal distress [216, 217], suggesting potential limitations in its applicability across different bacterial targets and disease conditions. However, phage therapy presents a uniquely targeted strategy to alter the gut microbiome, promoting healthy aging through removing harmful species associated with chronic inflammation and age-related diseases. Future applications may include personalized phage cocktails to restore eubiosis and promote gut health in older adults. More clinical validations are, of course, required to translate these findings into preventative practices.

## Conclusion

The gut microbiome is a salient contributor to human health, with considerable evidence also supporting it as a marker and mediator of healthy aging. The gut microbiome undergoes compositional and functional changes throughout the human lifespan, showing life stage-dependent characteristics, such as a milk-oriented composition in infancy, a solid food- and hormone-driven diversification during puberty, a steady and complex homeostasis in adulthood, and finally a reconfigured composition with increased uniqueness in the elderly, which is associated with healthy aging. Studies in model organisms have demonstrated that the gut microbiome affects aging and longevity through metabolic activities that modulate host immunity. This modulation is also present in the centenarian gut microbiomes, with unique characteristics that plausibly contribute to longevity, including increased microbial and metabolic diversity, enriched beneficial taxa like *Akkermansia* and Christensenellaceae, and enhanced gut homeostasis.

Mechanistically, the gut microbiome orchestrates the aging process through various pathways. These pathways change with age, with age-related gut dysbiosis reciprocally promoting inflammaging through the decreased production of anti-inflammatory SCFAs and declined gut barrier integrity. The worsened inflammation amplifies neuroinflammatory responses, triggering cognitive decline through the gut-brain axis. Furthermore, gut dysbiosis also negatively affect muscle mass and function, which in turn exacerbate frailty in the elderly.

Targeting the gut microbiome to promote health (and healthy aging) is not a new concept, but has yet to receive enough attention to clearly identify relationships and pathways within the human body. However, the results clearly indicate that diets high in fiber or those following a Mediterranean style are effective in remodeling gut microbiomes that are associated with improved health in older adults. While caloric restriction shows potential benefits in extending lifespan, the practice in the elderly should also consider the risk of frailty caused by muscle loss before being implemented. Regular exercise benefits human health by enhancing muscle-organ crosstalk and improving the gut microbiome across all ages; however, in older adults, regular moderate exercise is recommended to avoid the temporary disruptions to gut homeostasis caused by high-intensity exercise while supporting the optimization of the gut microbiome. Given the heterogeneity in probiotic strains, dosages, durations, symptoms, and endpoints of clinical trials, the beneficial effects of probiotic supplementation in the elderly remain context-dependent. One potential constraint in the host contributing to this variability is the reduced plasticity of the aged gut microbiome, suggesting that sustained intervention in the elderly may be necessary to achieve meaningful outcomes. Lastly, prebiotics, postbiotics, and phage therapy are rising microbiome-targeted approaches to support gut health in aging, with most findings currently based on preclinical models; thus, further clinical trials are needed to validate their safety and efficacy for precision interventions in older populations.

Some intriguing questions remain open for investigation. First, the human aging process is composed of nonlinear waves in molecular changes, with approximately 44 and 60 years of age being the two critical periods characterized by the highest number of dysregulated molecules and microbes [218]. These two chronological ages are therefore of research interest, warranting further investigation into strategies based on gut microbiome modulation that could mitigate dysregulation to slow down or, at the very least, alleviate the aging process and reduce disease risk in later life. Second, some microbially derived metabolites, such as phenylacetylglutamine [219], accelerate host cellular senescence. Identifying gut microbes driving these metabolic processes and targeting them through dietary interventions to reduce the substrates fueling these pathways could provide internal benefits to the aging process. Third, while SCFAs produced by gut bacteria have neuroprotective effects, their detrimental properties have also been observed in neurodegenerative models. Therefore, SCFAs may support brain health under healthy or pre-disease conditions but accelerate disease progression if neurodegeneration is underway. The dualistic nature of SCFAs in aging-related neurodegenerative diseases not only exemplifies the saying “prevention is better than cure,” but also emphasizes the need to further investigate the timing and context of SCFA-mediated interventions for healthy aging. Finally, considering the trending interest in live biotherapeutic products [220], identifying novel strains associated with long-lived populations (e.g., centenarians) may expand the conventional list of probiotics, such as *Bifidobacterium* and *Lactobacillus* species, thereby enabling more targeted probiotic interventions to promote healthy aging and ultimately longevity.

The relationships between alterations of the gut microbiome and the aging process exhibit a classic “chicken-and-egg” problem, where the gut microbiome and aging may determine the other in a complicated manner and thus make it challenging to establish causal roles. However, if they are inherently and continuously interwoven and constantly affecting each other, understanding changes in the aging-related gut microbiome provides a framework for modifying the aging process itself. By modulating the gut microbiome, we may be able to adjust the pace or trajectory of aging, representing a promising area of inquiry.

## Data Availability

No datasets were generated or analysed during the current study.

## References

[1] Partridge L, Deelen J, Slagboom PE. Facing up to the global challenges of ageing. Nature. 2018;561:45–56. 30185958 10.1038/s41586-018-0457-8

[2] Collaborators GBDD. Global age-sex-specific fertility, mortality, healthy life expectancy (HALE), and population estimates in 204 countries and territories, 1950–2019: a comprehensive demographic analysis for the Global Burden of Disease Study 2019. Lancet. 2020;396:1160–203. 33069325 10.1016/S0140-6736(20)30977-6 PMC7566045

[3] Harper S. Economic and social implications of aging societies. Science. 2014;346:587–91. 25359967 10.1126/science.1254405

[4] Lopez-Otin C, Blasco MA, Partridge L, Serrano M, Kroemer G. The hallmarks of aging. Cell. 2013;153:1194–217. 23746838 10.1016/j.cell.2013.05.039 PMC3836174

[5] Guerville F, De Souto BP, Ader I, Andrieu S, Casteilla L, Dray C, et al. Revisiting the hallmarks of aging to identify markers of biological age. J Prev Alzheimers Dis. 2020;7:56–64. 32010927 10.14283/jpad.2019.50

[6] Carter CS. A “gut feeling” to create a 10th hallmark of aging. J Gerontol A Biol Sci Med Sci. 2021;76:1891–4. 34245264 10.1093/gerona/glab191 PMC8825216

[7] Hou K, Wu ZX, Chen XY, Wang JQ, Zhang D, Xiao C, et al. Microbiota in health and diseases. Signal Transduct Target Ther. 2022;7:135. 35461318 10.1038/s41392-022-00974-4 PMC9034083

[8] de Vos WM, Tilg H, Van Hul M, Cani PD. Gut microbiome and health: mechanistic insights. Gut. 2022;71:1020–32. 35105664 10.1136/gutjnl-2021-326789 PMC8995832

[9] O’Hara AM, Shanahan F. The gut flora as a forgotten organ. EMBO Rep. 2006;7:688–93. 16819463 10.1038/sj.embor.7400731 PMC1500832

[10] O’Toole PW, Jeffery IB. Gut microbiota and aging. Science. 2015;350:1214–5. 26785481 10.1126/science.aac8469

[11] Buford TW. (Dis)trust your gut: the gut microbiome in age-related inflammation, health, and disease. Microbiome. 2017;5:80. 28709450 10.1186/s40168-017-0296-0 PMC5512975

[12] Kundu P, Blacher E, Elinav E, Pettersson S. Our gut microbiome: the evolving inner self. Cell. 2017;171:1481–93. 29245010 10.1016/j.cell.2017.11.024

[13] Bradley E, Haran J. The human gut microbiome and aging. Gut Microbes. 2024;16:2359677. 38831607 10.1080/19490976.2024.2359677 PMC11152108

[14] Ghosh TS, Shanahan F, O’Toole PW. The gut microbiome as a modulator of healthy ageing. Nat Rev Gastroenterol Hepatol. 2022;19:565–84. 35468952 10.1038/s41575-022-00605-x PMC9035980

[15] Walrath T, Dyamenahalli KU, Hulsebus HJ, McCullough RL, Idrovo JP, Boe DM, et al. Age-related changes in intestinal immunity and the microbiome. J Leukoc Biol. 2021;109:1045–61. 33020981 10.1002/JLB.3RI0620-405RR PMC8139861

[16] Biragyn A, Ferrucci L. Gut dysbiosis: a potential link between increased cancer risk in ageing and inflammaging. Lancet Oncol. 2018;19:e295–304. 29893261 10.1016/S1470-2045(18)30095-0 PMC6047065

[17] Smith P, Willemsen D, Popkes M, Metge F, Gandiwa E, Reichard M, et al. Regulation of life span by the gut microbiota in the short-lived African turquoise killifish. Elife. 2017;6:e27014. 28826469 10.7554/eLife.27014 PMC5566455

[18] Thevaranjan N, Puchta A, Schulz C, Naidoo A, Szamosi JC, Verschoor CP, et al. Age-associated microbial dysbiosis promotes intestinal permeability, systemic inflammation, and macrophage dysfunction. Cell Host Microbe. 2017;21:455-466.e4. 28407483 10.1016/j.chom.2017.03.002 PMC5392495

[19] Barcena C, Valdes-Mas R, Mayoral P, Garabaya C, Durand S, Rodriguez F, et al. Healthspan and lifespan extension by fecal microbiota transplantation into progeroid mice. Nat Med. 2019;25:1234–42. 31332389 10.1038/s41591-019-0504-5

[20] Chen Y, Zhang S, Zeng B, Zhao J, Yang M, Zhang M, et al. Transplant of microbiota from long-living people to mice reduces aging-related indices and transfers beneficial bacteria. Aging (Albany NY). 2020;12:4778–93. 32176868 10.18632/aging.102872 PMC7138539

[21] Jing Y, Wang Q, Bai F, Li Z, Li Y, Liu W, et al. Age-related alterations in gut homeostasis are microbiota dependent. NPJ Biofilms Microbiomes. 2025;11:51. 40133348 10.1038/s41522-025-00677-y PMC11937415

[22] Best L, Dost T, Esser D, Flor S, Gamarra AM, Haase M, et al. Metabolic modelling reveals the aging-associated decline of host-microbiome metabolic interactions in mice. Nat Microbiol. 2025;10:973–91. 40140706 10.1038/s41564-025-01959-z PMC11964932

[23] Virk B, Correia G, Dixon DP, Feyst I, Jia J, Oberleitner N, et al. Excessive folate synthesis limits lifespan in the *C. elegans*: *E. coli* aging model. BMC Biol. 2012;10:67. 22849329 10.1186/1741-7007-10-67 PMC3583181

[24] Han B, Sivaramakrishnan P, Lin CJ, Neve IAA, He J, Tay LWR, et al. Microbial genetic composition tunes host longevity. Cell. 2017;169:1249-1262.e13. 28622510 10.1016/j.cell.2017.05.036 PMC5635830

[25] Gusarov I, Gautier L, Smolentseva O, Shamovsky I, Eremina S, Mironov A, et al. Bacterial nitric oxide extends the lifespan of *C. elegans*. Cell. 2013;152:818–30. 23415229 10.1016/j.cell.2012.12.043

[26] Kim J, Jo Y, Lim G, Ji Y, Roh JH, Kim WG, et al. A microbiota-derived metabolite, 3-phenyllactic acid, prolongs healthspan by enhancing mitochondrial function and stress resilience via SKN-1/ATFS-1 in *C. elegans*. Nat Commun. 2024;15:10773. 39737960 10.1038/s41467-024-55015-1 PMC11686233

[27] Brummel T, Ching A, Seroude L, Simon AF, Benzer S. *Drosophila* lifespan enhancement by exogenous bacteria. Proc Natl Acad Sci U S A. 2004;101:12974–9. 15322271 10.1073/pnas.0405207101 PMC516503

[28] Ren C, Webster P, Finkel SE, Tower J. Increased internal and external bacterial load during *Drosophila* aging without life-span trade-off. Cell Metab. 2007;6:144–52. 17681150 10.1016/j.cmet.2007.06.006

[29] Keebaugh ES, Yamada R, Ja WW. The nutritional environment influences the impact of microbes on *Drosophila melanogaster* life span. MBio. 2019;10:e00885–19. 31289176 10.1128/mBio.00885-19 PMC6747722

[30] Obata F, Fons CO, Gould AP. Early-life exposure to low-dose oxidants can increase longevity via microbiome remodelling in *Drosophila*. Nat Commun. 2018;9:975. 29515102 10.1038/s41467-018-03070-w PMC5841413

[31] Guo L, Karpac J, Tran SL, Jasper H. PGRP-SC2 promotes gut immune homeostasis to limit commensal dysbiosis and extend lifespan. Cell. 2014;156:109–22. 24439372 10.1016/j.cell.2013.12.018 PMC3928474

[32] Li H, Qi Y, Jasper H. Preventing age-related decline of gut compartmentalization limits microbiota dysbiosis and extends lifespan. Cell Host Microbe. 2016;19:240–53. 26867182 10.1016/j.chom.2016.01.008 PMC5106289

[33] Shukla AK, Johnson K, Giniger E. Common features of aging fail to occur in *Drosophila* raised without a bacterial microbiome. iScience. 2021;24:102703. 34235409 10.1016/j.isci.2021.102703 PMC8246586

[34] Satokari R, Gronroos T, Laitinen K, Salminen S, Isolauri E. *Bifidobacterium* and *lactobacillus* DNA in the human placenta. Lett Appl Microbiol. 2009;48:8–12. 19018955 10.1111/j.1472-765X.2008.02475.x

[35] Aagaard K, Ma J, Antony KM, Ganu R, Petrosino J, Versalovic J. The placenta harbors a unique microbiome. Sci Transl Med. 2014;6:237ra265. 10.1126/scitranslmed.3008599 PMC4929217 24848255

[36] Rautava S, Collado MC, Salminen S, Isolauri E. Probiotics modulate host-microbe interaction in the placenta and fetal gut: a randomized, double-blind, placebo-controlled trial. Neonatology. 2012;102:178–84. 22776980 10.1159/000339182

[37] Jimenez E, Fernandez L, Marin ML, Martin R, Odriozola JM, Nueno-Palop C, et al. Isolation of commensal bacteria from umbilical cord blood of healthy neonates born by cesarean section. Curr Microbiol. 2005;51:270–4. 16187156 10.1007/s00284-005-0020-3

[38] Younge N, McCann JR, Ballard J, Plunkett C, Akhtar S, Araujo-Perez F, et al. Fetal exposure to the maternal microbiota in humans and mice. JCI Insight. 2019;4:e127806. 31479427 10.1172/jci.insight.127806 PMC6795398

[39] Stinson LF, Payne MS, Keelan JA. Planting the seed: origins, composition, and postnatal health significance of the fetal gastrointestinal microbiota. Crit Rev Microbiol. 2017;43:352–69. 27931152 10.1080/1040841X.2016.1211088

[40] de Goffau MC, Lager S, Sovio U, Gaccioli F, Cook E, Peacock SJ, et al. Human placenta has no microbiome but can contain potential pathogens. Nature. 2019;572:329–34. 31367035 10.1038/s41586-019-1451-5 PMC6697540

[41] Perez-Munoz ME, Arrieta MC, Ramer-Tait AE, Walter J. A critical assessment of the “sterile womb” and “in utero colonization” hypotheses: implications for research on the pioneer infant microbiome. Microbiome. 2017;5:48. 28454555 10.1186/s40168-017-0268-4 PMC5410102

[42] Kennedy KM, de Goffau MC, Perez-Munoz ME, Arrieta MC, Backhed F, Bork P, et al. Questioning the fetal microbiome illustrates pitfalls of low-biomass microbial studies. Nature. 2023;613:639–49. 36697862 10.1038/s41586-022-05546-8 PMC11333990

[43] Dominguez-Bello MG, Costello EK, Contreras M, Magris M, Hidalgo G, Fierer N, et al. Delivery mode shapes the acquisition and structure of the initial microbiota across multiple body habitats in newborns. Proc Natl Acad Sci U S A. 2010;107:11971–5. 20566857 10.1073/pnas.1002601107 PMC2900693

[44] Bager P, Wohlfahrt J, Westergaard T. Caesarean delivery and risk of atopy and allergic disease: meta-analyses. Clin Exp Allergy. 2008;38:634–42. 18266879 10.1111/j.1365-2222.2008.02939.x

[45] Olszak T, An D, Zeissig S, Vera MP, Richter J, Franke A, et al. Microbial exposure during early life has persistent effects on natural killer T cell function. Science. 2012;336:489–93. 22442383 10.1126/science.1219328 PMC3437652

[46] Arrieta MC, Stiemsma LT, Dimitriu PA, Thorson L, Russell S, Yurist-Doutsch S, et al. Early infancy microbial and metabolic alterations affect risk of childhood asthma. Sci Transl Med. 2015;7:307ra152. 26424567 10.1126/scitranslmed.aab2271

[47] Stokholm J, Thorsen J, Blaser MJ, Rasmussen MA, Hjelmso M, Shah S, et al. Delivery mode and gut microbial changes correlate with an increased risk of childhood asthma. Sci Transl Med. 2020;12:eaax9929. 33177184 10.1126/scitranslmed.aax9929

[48] Hoskinson C, Dai DLY, Del Bel KL, Becker AB, Moraes TJ, Mandhane PJ, et al. Delayed gut microbiota maturation in the first year of life is a hallmark of pediatric allergic disease. Nat Commun. 2023;14:4785. 37644001 10.1038/s41467-023-40336-4 PMC10465508

[49] Martin R, Langa S, Reviriego C, Jiminez E, Marin ML, Xaus J, et al. Human milk is a source of lactic acid bacteria for the infant gut. J Pediatr. 2003;143:754–8. 14657823 10.1016/j.jpeds.2003.09.028

[50] Fernandez L, Langa S, Martin V, Maldonado A, Jimenez E, Martin R, et al. The human milk microbiota: origin and potential roles in health and disease. Pharmacol Res. 2013;69:1–10. 22974824 10.1016/j.phrs.2012.09.001

[51] Heikkila MP, Saris PE. Inhibition of *Staphylococcus aureus* by the commensal bacteria of human milk. J Appl Microbiol. 2003;95:471–8. 12911694 10.1046/j.1365-2672.2003.02002.x

[52] Collado MC, Delgado S, Maldonado A, Rodriguez JM. Assessment of the bacterial diversity of breast milk of healthy women by quantitative real-time PCR. Lett Appl Microbiol. 2009;48:523–8. 19228290 10.1111/j.1472-765X.2009.02567.x

[53] Hunt KM, Foster JA, Forney LJ, Schutte UM, Beck DL, Abdo Z, et al. Characterization of the diversity and temporal stability of bacterial communities in human milk. PLoS ONE. 2011;6:e21313. 21695057 10.1371/journal.pone.0021313 PMC3117882

[54] Cabrera-Rubio R, Collado MC, Laitinen K, Salminen S, Isolauri E, Mira A. The human milk microbiome changes over lactation and is shaped by maternal weight and mode of delivery. Am J Clin Nutr. 2012;96:544–51. 22836031 10.3945/ajcn.112.037382

[55] Moossavi S, Sepehri S, Robertson B, Bode L, Goruk S, Field CJ, et al. Composition and variation of the human milk microbiota are influenced by maternal and early-life factors. Cell Host Microbe. 2019;25:324-335.e4. 30763539 10.1016/j.chom.2019.01.011

[56] Bode L. Human milk oligosaccharides: every baby needs a sugar mama. Glycobiology. 2012;22:1147–62. 22513036 10.1093/glycob/cws074 PMC3406618

[57] Azad MB, Konya T, Maughan H, Guttman DS, Field CJ, Chari RS, et al. Gut microbiota of healthy Canadian infants: profiles by mode of delivery and infant diet at 4 months. CMAJ. 2013;185:385–94. 23401405 10.1503/cmaj.121189 PMC3602254

[58] Henrick BM, Rodriguez L, Lakshmikanth T, Pou C, Henckel E, Arzoomand A, et al. Bifidobacteria-mediated immune system imprinting early in life. Cell. 2021;184:3884-3898.e11. 34143954 10.1016/j.cell.2021.05.030

[59] Hickman B, Salonen A, Ponsero AJ, Jokela R, Kolho KL, de Vos WM, et al. Gut microbiota wellbeing index predicts overall health in a cohort of 1000 infants. Nat Commun. 2024;15:8323. 39333099 10.1038/s41467-024-52561-6 PMC11436675

[60] Backhed F, Roswall J, Peng Y, Feng Q, Jia H, Kovatcheva-Datchary P, et al. Dynamics and stabilization of the human gut microbiome during the first year of life. Cell Host Microbe. 2015;17:690–703. 25974306 10.1016/j.chom.2015.04.004

[61] Stewart CJ, Ajami NJ, O’Brien JL, Hutchinson DS, Smith DP, Wong MC, et al. Temporal development of the gut microbiome in early childhood from the TEDDY study. Nature. 2018;562:583–8. 30356187 10.1038/s41586-018-0617-x PMC6415775

[62] Yatsunenko T, Rey FE, Manary MJ, Trehan I, Dominguez-Bello MG, Contreras M, et al. Human gut microbiome viewed across age and geography. Nature. 2012;486:222–7. 22699611 10.1038/nature11053 PMC3376388

[63] Koenig JE, Spor A, Scalfone N, Fricker AD, Stombaugh J, Knight R, et al. Succession of microbial consortia in the developing infant gut microbiome. Proc Natl Acad Sci U S A. 2011;108(Suppl 1):4578–85. 20668239 10.1073/pnas.1000081107 PMC3063592

[64] Sawhney SS, Thanert R, Thanert A, Hall-Moore C, Ndao IM, Mahmud B, et al. Gut microbiome evolution from infancy to 8 years of age. Nat Med. 2025. 10.1038/s41591-025-03610-0. 40175737 10.1038/s41591-025-03610-0 PMC12302008

[65] Differding MK, Doyon M, Bouchard L, Perron P, Guerin R, Asselin C, et al. Potential interaction between timing of infant complementary feeding and breastfeeding duration in determination of early childhood gut microbiota composition and BMI. Pediatr Obes. 2020;15:e12642. 32351036 10.1111/ijpo.12642 PMC7923600

[66] Lalli MK, Salo TE, Hakola L, Knip M, Virtanen SM, Vatanen T. Associations between dietary fibers and gut microbiome composition in the EDIA longitudinal infant cohort. Am J Clin Nutr. 2025;121:83–99. 39551356 10.1016/j.ajcnut.2024.11.011 PMC11747200

[67] Agans R, Rigsbee L, Kenche H, Michail S, Khamis HJ, Paliy O. Distal gut microbiota of adolescent children is different from that of adults. FEMS Microbiol Ecol. 2011;77:404–12. 21539582 10.1111/j.1574-6941.2011.01120.x PMC4502954

[68] Hollister EB, Riehle K, Luna RA, Weidler EM, Rubio-Gonzales M, Mistretta TA, et al. Structure and function of the healthy pre-adolescent pediatric gut microbiome. Microbiome. 2015;3:36. 26306392 10.1186/s40168-015-0101-x PMC4550057

[69] Markle JG, Frank DN, Mortin-Toth S, Robertson CE, Feazel LM, Rolle-Kampczyk U, et al. Sex differences in the gut microbiome drive hormone-dependent regulation of autoimmunity. Science. 2013;339:1084–8. 23328391 10.1126/science.1233521

[70] Yurkovetskiy L, Burrows M, Khan AA, Graham L, Volchkov P, Becker L, et al. Gender bias in autoimmunity is influenced by microbiota. Immunity. 2013;39:400–12. 23973225 10.1016/j.immuni.2013.08.013 PMC3822899

[71] Weger BD, Gobet C, Yeung J, Martin E, Jimenez S, Betrisey B, et al. The mouse microbiome is required for sex-specific diurnal rhythms of gene expression and metabolism. Cell Metab. 2019;29:362-382.e8. 30344015 10.1016/j.cmet.2018.09.023 PMC6370974

[72] Korpela K, Kallio S, Salonen A, Hero M, Kukkonen AK, Miettinen PJ, et al. Gut microbiota develop towards an adult profile in a sex-specific manner during puberty. Sci Rep. 2021;11:23297. 34857814 10.1038/s41598-021-02375-z PMC8640005

[73] Wang L, Yi Q, Xu H, Liu H, Tan B, Deng H, et al. Alterations in the gut microbiota community are associated with childhood obesity and precocious puberty. BMC Microbiol. 2024;24:311. 39182062 10.1186/s12866-024-03461-8 PMC11344344

[74] Ou Y, Belzer C, Smidt H, de Weerth C. Development of the gut microbiota in the first 14 years of life and its relations to internalizing and externalizing difficulties and social anxiety during puberty. Eur Child Adolesc Psychiatry. 2024;33:847–60. 37071196 10.1007/s00787-023-02205-9 PMC10894087

[75] Murray E, Sharma R, Smith KB, Mar KD, Barve R, Lukasik M, et al. Probiotic consumption during puberty mitigates LPS-induced immune responses and protects against stress-induced depression- and anxiety-like behaviors in adulthood in a sex-specific manner. Brain Behav Immun. 2019;81:198–212. 31212008 10.1016/j.bbi.2019.06.016

[76] Human Microbiome Project C. Structure, function and diversity of the healthy human microbiome. Nature. 2012;486:207–14. 22699609 10.1038/nature11234 PMC3564958

[77] Lloyd-Price J, Mahurkar A, Rahnavard G, Crabtree J, Orvis J, Hall AB, et al. Strains, functions and dynamics in the expanded Human Microbiome Project. Nature. 2017;550:61–6. 28953883 10.1038/nature23889 PMC5831082

[78] McDonald D, Hyde E, Debelius JW, Morton JT, Gonzalez A, Ackermann G, et al. American gut: an open platform for citizen science microbiome research. mSystems. 2018;3:e00031-18. 29795809 10.1128/mSystems.00031-18 PMC5954204

[79] Qin J, Li R, Raes J, Arumugam M, Burgdorf KS, Manichanh C, et al. A human gut microbial gene catalogue established by metagenomic sequencing. Nature. 2010;464:59–65. 20203603 10.1038/nature08821 PMC3779803

[80] Li J, Jia H, Cai X, Zhong H, Feng Q, Sunagawa S, et al. An integrated catalog of reference genes in the human gut microbiome. Nat Biotechnol. 2014;32:834–41. 24997786 10.1038/nbt.2942

[81] Qin J, Li Y, Cai Z, Li S, Zhu J, Zhang F, et al. A metagenome-wide association study of gut microbiota in type 2 diabetes. Nature. 2012;490:55–60. 23023125 10.1038/nature11450

[82] Lu J, Zhang L, Zhai Q, Zhao J, Zhang H, Lee YK, et al. Chinese gut microbiota and its associations with staple food type, ethnicity, and urbanization. NPJ Biofilms Microbiomes. 2021;7:71. 34489454 10.1038/s41522-021-00245-0 PMC8421333

[83] Tian C, Zhang T, Zhuang D, Luo Y, Li T, Zhao F, et al. Industrialization drives the gut microbiome and resistome of the Chinese populations. mSystems. 2025;10:e0137224. 39902937 10.1128/msystems.01372-24 PMC11915869

[84] Asnicar F, Berry SE, Valdes AM, Nguyen LH, Piccinno G, Drew DA, et al. Microbiome connections with host metabolism and habitual diet from 1098 deeply phenotyped individuals. Nat Med. 2021;27:321–32. 33432175 10.1038/s41591-020-01183-8 PMC8353542

[85] Falony G, Joossens M, Vieira-Silva S, Wang J, Darzi Y, Faust K, et al. Population-level analysis of gut microbiome variation. Science. 2016;352:560–4. 27126039 10.1126/science.aad3503

[86] Zhernakova A, Kurilshikov A, Bonder MJ, Tigchelaar EF, Schirmer M, Vatanen T, et al. Population-based metagenomics analysis reveals markers for gut microbiome composition and diversity. Science. 2016;352:565–9. 27126040 10.1126/science.aad3369 PMC5240844

[87] Gacesa R, Kurilshikov A, Vich Vila A, Sinha T, Klaassen MAY, Bolte LA, et al. Environmental factors shaping the gut microbiome in a Dutch population. Nature. 2022;604:732–9. 35418674 10.1038/s41586-022-04567-7

[88] Nishijima S, Suda W, Oshima K, Kim SW, Hirose Y, Morita H, et al. The gut microbiome of healthy Japanese and its microbial and functional uniqueness. DNA Res. 2016;23:125–33. 26951067 10.1093/dnares/dsw002 PMC4833420

[89] Park J, Kato K, Murakami H, Hosomi K, Tanisawa K, Nakagata T, et al. Comprehensive analysis of gut microbiota of a healthy population and covariates affecting microbial variation in two large Japanese cohorts. BMC Microbiol. 2021;21:151. 34016052 10.1186/s12866-021-02215-0 PMC8139087

[90] Lozupone CA, Stombaugh JI, Gordon JI, Jansson JK, Knight R. Diversity, stability and resilience of the human gut microbiota. Nature. 2012;489:220–30. 22972295 10.1038/nature11550 PMC3577372

[91] Abdill RJ, Graham SP, Rubinetti V, Ahmadian M, Hicks P, Chetty A, et al. Integration of 168,000 samples reveals global patterns of the human gut microbiome. Cell. 2025;188:1100-1118.e17. 39848248 10.1016/j.cell.2024.12.017 PMC11848717

[92] Rosenberg E. Diversity of bacteria within the human gut and its contribution to the functional unity of holobionts. NPJ Biofilms Microbiomes. 2024;10:134. 39580487 10.1038/s41522-024-00580-y PMC11585559

[93] Sommer F, Anderson JM, Bharti R, Raes J, Rosenstiel P. The resilience of the intestinal microbiota influences health and disease. Nat Rev Microbiol. 2017;15:630–8. 28626231 10.1038/nrmicro.2017.58

[94] Ives AR, Carpenter SR. Stability and diversity of ecosystems. Science. 2007;317:58–62. 17615333 10.1126/science.1133258

[95] Mancabelli L, Milani C, De Biase R, Bocchio F, Fontana F, Lugli GA, et al. Taxonomic and metabolic development of the human gut microbiome across life stages: a worldwide metagenomic investigation. mSystems. 2024;9:e0129423. 38441032 10.1128/msystems.01294-23 PMC11019788

[96] Faith JJ, Guruge JL, Charbonneau M, Subramanian S, Seedorf H, Goodman AL, et al. The long-term stability of the human gut microbiota. Science. 2013;341:1237439. 23828941 10.1126/science.1237439 PMC3791589

[97] Safarchi A, Al-Qadami G, Tran CD, Conlon M. Understanding dysbiosis and resilience in the human gut microbiome: biomarkers, interventions, and challenges. Front Microbiol. 2025;16:1559521. 40104586 10.3389/fmicb.2025.1559521 PMC11913848

[98] Yassour M, Vatanen T, Siljander H, Hamalainen AM, Harkonen T, Ryhanen SJ, et al. Natural history of the infant gut microbiome and impact of antibiotic treatment on bacterial strain diversity and stability. Sci Transl Med. 2016;8:343ra381. 10.1126/scitranslmed.aad0917 PMC5032909 27306663

[99] Li X, Brejnrod A, Thorsen J, Zachariasen T, Trivedi U, Russel J, et al. Differential responses of the gut microbiome and resistome to antibiotic exposures in infants and adults. Nat Commun. 2023;14:8526. 38135681 10.1038/s41467-023-44289-6 PMC10746713

[100] Zaura E, Brandt BW, Teixeira de Mattos MJ, Buijs MJ, Caspers MP, Rashid MU, et al. Same exposure but two radically different responses to antibiotics: resilience of the salivary microbiome versus long-term microbial shifts in feces. MBio. 2015;6:e01693-01615. 26556275 10.1128/mBio.01693-15 PMC4659469

[101] Hildebrand F, Gossmann TI, Frioux C, Ozkurt E, Myers PN, Ferretti P, et al. Dispersal strategies shape persistence and evolution of human gut bacteria. Cell Host Microbe. 2021;29:1167-1176.e9. 34111423 10.1016/j.chom.2021.05.008 PMC8288446

[102] Olsson LM, Boulund F, Nilsson S, Khan MT, Gummesson A, Fagerberg L, et al. Dynamics of the normal gut microbiota: a longitudinal one-year population study in Sweden. Cell Host Microbe. 2022;30:726-739.e3. 35349787 10.1016/j.chom.2022.03.002

[103] Han N, Zhang T, Qiang Y, Peng X, Li X, Zhang W. Time-scale analysis of the long-term variability of human gut microbiota characteristics in Chinese individuals. Commun Biol. 2022;5:1414. 36564493 10.1038/s42003-022-04359-9 PMC9789056

[104] Goodrich JK, Waters JL, Poole AC, Sutter JL, Koren O, Blekhman R, et al. Human genetics shape the gut microbiome. Cell. 2014;159:789–99. 25417156 10.1016/j.cell.2014.09.053 PMC4255478

[105] Blekhman R, Goodrich JK, Huang K, Sun Q, Bukowski R, Bell JT, et al. Host genetic variation impacts microbiome composition across human body sites. Genome Biol. 2015;16:191. 26374288 10.1186/s13059-015-0759-1 PMC4570153

[106] Goodrich JK, Davenport ER, Beaumont M, Jackson MA, Knight R, Ober C, et al. Genetic determinants of the gut microbiome in UK twins. Cell Host Microbe. 2016;19:731–43. 27173935 10.1016/j.chom.2016.04.017 PMC4915943

[107] Bonder MJ, Kurilshikov A, Tigchelaar EF, Mujagic Z, Imhann F, Vila AV, et al. The effect of host genetics on the gut microbiome. Nat Genet. 2016;48:1407–12. 27694959 10.1038/ng.3663

[108] Ruhlemann MC, Hermes BM, Bang C, Doms S, Moitinho-Silva L, Thingholm LB, et al. Genome-wide association study in 8956 German individuals identifies influence of ABO histo-blood groups on gut microbiome. Nat Genet. 2021;53:147–55. 33462482 10.1038/s41588-020-00747-1

[109] Lopera-Maya EA, Kurilshikov A, van der Graaf A, Hu S, Andreu-Sanchez S, Chen L, et al. Effect of host genetics on the gut microbiome in 7738 participants of the Dutch Microbiome Project. Nat Genet. 2022;54:143–51. 35115690 10.1038/s41588-021-00992-y

[110] Qin Y, Havulinna AS, Liu Y, Jousilahti P, Ritchie SC, Tokolyi A, et al. Combined effects of host genetics and diet on human gut microbiota and incident disease in a single population cohort. Nat Genet. 2022;54:134–42. 35115689 10.1038/s41588-021-00991-z PMC9883041

[111] Wang J, Thingholm LB, Skieceviciene J, Rausch P, Kummen M, Hov JR, et al. Genome-wide association analysis identifies variation in vitamin D receptor and other host factors influencing the gut microbiota. Nat Genet. 2016;48:1396–406. 27723756 10.1038/ng.3695 PMC5626933

[112] Liu X, Tang S, Zhong H, Tong X, Jie Z, Ding Q, et al. A genome-wide association study for gut metagenome in Chinese adults illuminates complex diseases. Cell Discov. 2021;7:9. 33563976 10.1038/s41421-020-00239-w PMC7873036

[113] Rothschild D, Weissbrod O, Barkan E, Kurilshikov A, Korem T, Zeevi D, et al. Environment dominates over host genetics in shaping human gut microbiota. Nature. 2018;555:210–5. 29489753 10.1038/nature25973

[114] Barker-Tejeda TC, Zubeldia-Varela E, Macias-Camero A, Alonso L, Martin-Antoniano IA, Rey-Stolle MF, et al. Comparative characterization of the infant gut microbiome and their maternal lineage by a multi-omics approach. Nat Commun. 2024;15:3004. 38589361 10.1038/s41467-024-47182-y PMC11001937

[115] Biagi E, Nylund L, Candela M, Ostan R, Bucci L, Pini E, et al. Through ageing, and beyond: Gut microbiota and inflammatory status in seniors and centenarians. PLoS ONE. 2010;5:e10667. 20498852 10.1371/journal.pone.0010667 PMC2871786

[116] Rampelli S, Candela M, Turroni S, Biagi E, Collino S, Franceschi C, et al. Functional metagenomic profiling of intestinal microbiome in extreme ageing. Aging (Albany NY). 2013;5:902–12. 24334635 10.18632/aging.100623 PMC3883706

[117] Arthur JC, Perez-Chanona E, Muhlbauer M, Tomkovich S, Uronis JM, Fan TJ, et al. Intestinal inflammation targets cancer-inducing activity of the microbiota. Sci. 2012;338:120–3. 10.1126/science.1224820 PMC3645302 22903521

[118] Cougnoux A, Dalmasso G, Martinez R, Buc E, Delmas J, Gibold L, et al. Bacterial genotoxin colibactin promotes colon tumour growth by inducing a senescence-associated secretory phenotype. Gut. 2014;63:1932–42. 24658599 10.1136/gutjnl-2013-305257

[119] Yu LC, Wei SC, Li YH, Lin PY, Chang XY, Weng JP, et al. Invasive pathobionts contribute to colon cancer initiation by counterbalancing epithelial antimicrobial responses. Cell Mol Gastroenterol Hepatol. 2022;13:57–79. 34418587 10.1016/j.jcmgh.2021.08.007 PMC8600093

[120] Pai YC, Li YH, Turner JR, Yu LC. Transepithelial barrier dysfunction drives microbiota dysbiosis to initiate epithelial clock-driven inflammation. J Crohns Colitis. 2023;17:1471–88. 37004200 10.1093/ecco-jcc/jjad064 PMC10588795

[121] Claesson MJ, Cusack S, O’Sullivan O, Greene-Diniz R, de Weerd H, Flannery E, et al. Composition, variability, and temporal stability of the intestinal microbiota of the elderly. Proc Natl Acad Sci U S A. 2011;108(Suppl 1):4586–91. 20571116 10.1073/pnas.1000097107 PMC3063589

[122] Claesson MJ, Jeffery IB, Conde S, Power SE, O’Connor EM, Cusack S, et al. Gut microbiota composition correlates with diet and health in the elderly. Nature. 2012;488:178–84. 22797518 10.1038/nature11319

[123] Jeffery IB, Lynch DB, O’Toole PW. Composition and temporal stability of the gut microbiota in older persons. ISME J. 2016;10:170–82. 26090993 10.1038/ismej.2015.88 PMC4681863

[124] Kheirbek RE, Fokar A, Shara N, Bell-Wilson LK, Moore HJ, Olsen E, et al. Characteristics and incidence of chronic illness in community-dwelling predominantly male US veteran centenarians. J Am Geriatr Soc. 2017;65:2100–6. 28422270 10.1111/jgs.14900 PMC5724032

[125] Biagi E, Franceschi C, Rampelli S, Severgnini M, Ostan R, Turroni S, et al. Gut microbiota and extreme longevity. Curr Biol. 2016;26:1480–5. 27185560 10.1016/j.cub.2016.04.016

[126] Kong F, Hua Y, Zeng B, Ning R, Li Y, Zhao J. Gut microbiota signatures of longevity. Curr Biol. 2016;26:R832–3. 27676296 10.1016/j.cub.2016.08.015

[127] Wang F, Yu T, Huang G, Cai D, Liang X, Su H, et al. Gut microbiota community and its assembly associated with age and diet in Chinese centenarians. J Microbiol Biotechnol. 2015;25:1195–204. 25839332 10.4014/jmb.1410.10014

[128] Odamaki T, Kato K, Sugahara H, Hashikura N, Takahashi S, Xiao JZ, et al. Age-related changes in gut microbiota composition from newborn to centenarian: a cross-sectional study. BMC Microbiol. 2016;16:90. 27220822 10.1186/s12866-016-0708-5 PMC4879732

[129] Santoro A, Ostan R, Candela M, Biagi E, Brigidi P, Capri M, et al. Gut microbiota changes in the extreme decades of human life: a focus on centenarians. Cell Mol Life Sci. 2018;75:129–48. 29032502 10.1007/s00018-017-2674-y PMC5752746

[130] Pang S, Chen X, Lu Z, Meng L, Huang Y, Yu X, et al. Longevity of centenarians is reflected by the gut microbiome with youth-associated signatures. Nat Aging. 2023;3:436–49. 37117794 10.1038/s43587-023-00389-y

[131] Wilmanski T, Diener C, Rappaport N, Patwardhan S, Wiedrick J, Lapidus J, et al. Gut microbiome pattern reflects healthy ageing and predicts survival in humans. Nat Metab. 2021;3:274–86. 33619379 10.1038/s42255-021-00348-0 PMC8169080

[132] Ghosh TS, Shanahan F, O’Toole PW. Toward an improved definition of a healthy microbiome for healthy aging. Nat Aging. 2022;2:1054–69. 37118093 10.1038/s43587-022-00306-9 PMC10154212

[133] Cheng S, Larson MG, McCabe EL, Murabito JM, Rhee EP, Ho JE, et al. Distinct metabolomic signatures are associated with longevity in humans. Nat Commun. 2015;6(1):6791. 25864806 10.1038/ncomms7791 PMC4396657

[134] Wu L, Zeng T, Zinellu A, Rubino S, Kelvin DJ, Carru C. A cross-sectional study of compositional and functional profiles of gut microbiota in Sardinian centenarians. mSystems. 2019;4:e00325–19. 31289141 10.1128/mSystems.00325-19 PMC6616150

[135] Wu L, Xie X, Li Y, Liang T, Zhong H, Yang L, et al. Gut microbiota as an antioxidant system in centenarians associated with high antioxidant activities of gut-resident *Lactobacillus*. NPJ Biofilms Microbiomes. 2022;8:102. 36564415 10.1038/s41522-022-00366-0 PMC9789086

[136] Sato Y, Atarashi K, Plichta DR, Arai Y, Sasajima S, Kearney SM, et al. Novel bile acid biosynthetic pathways are enriched in the microbiome of centenarians. Nature. 2021;599:458–64. 34325466 10.1038/s41586-021-03832-5

[137] Liu S, Zhang Z, Wang X, Ma Y, Ruan H, Wu X, et al. Biosynthetic potential of the gut microbiome in longevous populations. Gut Microbes. 2024;16:2426623. 39529240 10.1080/19490976.2024.2426623 PMC11559365

[138] Si J, Vazquez-Castellanos JF, Gregory AC, Decommer L, Rymenans L, Proost S, et al. Long-term life history predicts current gut microbiome in a population-based cohort study. Nat Aging. 2022;2:885–95. 37118287 10.1038/s43587-022-00286-w PMC10154234

[139] Franceschi C, Campisi J. Chronic inflammation (inflammaging) and its potential contribution to age-associated diseases. J Gerontol A Biol Sci Med Sci. 2014;69(Suppl 1):S4-9. 24833586 10.1093/gerona/glu057

[140] Nikolich-Zugich J. The twilight of immunity: emerging concepts in aging of the immune system. Nat Immunol. 2018;19:10–9. 29242543 10.1038/s41590-017-0006-x

[141] Bosco N, Noti M. The aging gut microbiome and its impact on host immunity. Genes Immun. 2021;22:289–303. 33875817 10.1038/s41435-021-00126-8 PMC8054695

[142] Correa-Oliveira R, Fachi JL, Vieira A, Sato FT, Vinolo MA. Regulation of immune cell function by short-chain fatty acids. Clin Transl Immunol. 2016;5:e73. 10.1038/cti.2016.17 PMC4855267 27195116

[143] Louis P, Flint HJ. Diversity, metabolism and microbial ecology of butyrate-producing bacteria from the human large intestine. FEMS Microbiol Lett. 2009;294:1–8. 19222573 10.1111/j.1574-6968.2009.01514.x

[144] Maslowski KM, Vieira AT, Ng A, Kranich J, Sierro F, Yu D, et al. Regulation of inflammatory responses by gut microbiota and chemoattractant receptor GPR43. Nature. 2009;461:1282–6. 19865172 10.1038/nature08530 PMC3256734

[145] Cryan JF, O’Riordan KJ, Cowan CSM, Sandhu KV, Bastiaanssen TFS, Boehme M, et al. The microbiota-gut-brain axis. Physiol Rev. 2019;99:1877–2013. 31460832 10.1152/physrev.00018.2018

[146] Solanki R, Karande A, Ranganathan P. Emerging role of gut microbiota dysbiosis in neuroinflammation and neurodegeneration. Front Neurol. 2023;14:1149618. 37255721 10.3389/fneur.2023.1149618 PMC10225576

[147] Rothhammer V, Borucki DM, Tjon EC, Takenaka MC, Chao CC, Ardura-Fabregat A, et al. Microglial control of astrocytes in response to microbial metabolites. Nature. 2018;557:724–8. 29769726 10.1038/s41586-018-0119-x PMC6422159

[148] Erny D, Hrabe de Angelis AL, Jaitin D, Wieghofer P, Staszewski O, David E, et al. Host microbiota constantly control maturation and function of microglia in the CNS. Nat Neurosci. 2015;18:965–77. 26030851 10.1038/nn.4030 PMC5528863

[149] Erny D, Dokalis N, Mezo C, Castoldi A, Mossad O, Staszewski O, et al. Microbiota-derived acetate enables the metabolic fitness of the brain innate immune system during health and disease. Cell Metab. 2021;33:2260-2276.e7. 34731656 10.1016/j.cmet.2021.10.010

[150] Duscha A, Gisevius B, Hirschberg S, Yissachar N, Stangl GI, Dawin E, et al. Propionic acid shapes the multiple sclerosis disease course by an immunomodulatory mechanism. Cell. 2020;180:1067-1080.e6. 32160527 10.1016/j.cell.2020.02.035

[151] Liu X, Li X, Xia B, Jin X, Zou Q, Zeng Z, et al. High-fiber diet mitigates maternal obesity-induced cognitive and social dysfunction in the offspring via gut-brain axis. Cell Metab. 2021;33:923-938.e6. 33651981 10.1016/j.cmet.2021.02.002

[152] Seo DO, O’Donnell D, Jain N, Ulrich JD, Herz J, Li Y, et al. ApoE isoform- and microbiota-dependent progression of neurodegeneration in a mouse model of tauopathy. Science. 2023;379:eadd1236. 36634180 10.1126/science.add1236 PMC9901565

[153] Colombo AV, Sadler RK, Llovera G, Singh V, Roth S, Heindl S, et al. Microbiota-derived short chain fatty acids modulate microglia and promote Abeta plaque deposition. Elife. 2021;10:e59826. 33845942 10.7554/eLife.59826 PMC8043748

[154] Sampson TR, Debelius JW, Thron T, Janssen S, Shastri GG, Ilhan ZE, et al. Gut microbiota regulate motor deficits and neuroinflammation in a model of Parkinson’s disease. Cell. 2016;167(1469–1480):e1412. 10.1016/j.cell.2016.11.018 PMC5718049 27912057

[155] Shin C, Lim Y, Lim H, Ahn TB. Plasma short-chain fatty acids in patients with Parkinson’s disease. Mov Disord. 2020;35:1021–7. 32154946 10.1002/mds.28016

[156] Yang X, Ai P, He X, Mo C, Zhang Y, Xu S, et al. Parkinson’s disease is associated with impaired gut-blood barrier for short-chain fatty acids. Mov Disord. 2022;37:1634–43. 35607987 10.1002/mds.29063

[157] Jackson MA, Jeffery IB, Beaumont M, Bell JT, Clark AG, Ley RE, et al. Signatures of early frailty in the gut microbiota. Genome Med. 2016;8:8. 26822992 10.1186/s13073-016-0262-7 PMC4731918

[158] Almeida HM, Sardeli AV, Conway J, Duggal NA, Cavaglieri CR. Comparison between frail and non-frail older adults’ gut microbiota: a systematic review and meta-analysis. Ageing Res Rev. 2022;82:101773. 36349647 10.1016/j.arr.2022.101773

[159] Lahiri S, Kim H, Garcia-Perez I, Reza MM, Martin KA, Kundu P, et al. The gut microbiota influences skeletal muscle mass and function in mice. Sci Transl Med. 2019;11:eaan5662. 31341063 10.1126/scitranslmed.aan5662 PMC7501733

[160] Li G, Jin B, Fan Z. Mechanisms involved in gut microbiota regulation of skeletal muscle. Oxid Med Cell Longev. 2022;2022:2151191. 35633886 10.1155/2022/2151191 PMC9132697

[161] Han DS, Wu WK, Liu PY, Yang YT, Hsu HC, Kuo CH, et al. Differences in the gut microbiome and reduced fecal butyrate in elders with low skeletal muscle mass. Clin Nutr. 2022;41:1491–500. 35667265 10.1016/j.clnu.2022.05.008

[162] Otsuka R, Zhang S, Furuya K, Tange C, Sala G, Ando F, et al. Association between short-chain fatty acid intake and development of muscle strength loss among community-dwelling older Japanese adults. Exp Gerontol. 2023;173:112080. 36634721 10.1016/j.exger.2023.112080

[163] Liu C, Wong PY, Wang Q, Wong HY, Huang T, Cui C, et al. Short-chain fatty acids enhance muscle mass and function through the activation of mTOR signalling pathways in sarcopenic mice. J Cachexia Sarcopenia Muscle. 2024;15:2387–401. 39482890 10.1002/jcsm.13573 PMC11634463

[164] Wu GD, Chen J, Hoffmann C, Bittinger K, Chen YY, Keilbaugh SA, et al. Linking long-term dietary patterns with gut microbial enterotypes. Science. 2011;334:105–8. 21885731 10.1126/science.1208344 PMC3368382

[165] David LA, Maurice CF, Carmody RN, Gootenberg DB, Button JE, Wolfe BE, et al. Diet rapidly and reproducibly alters the human gut microbiome. Nature. 2014;505:559–63. 24336217 10.1038/nature12820 PMC3957428

[166] Fackelmann G, Manghi P, Carlino N, Heidrich V, Piccinno G, Ricci L, et al. Gut microbiome signatures of vegan, vegetarian and omnivore diets and associated health outcomes across 21,561 individuals. Nat Microbiol. 2025;10:41–52. 39762435 10.1038/s41564-024-01870-z PMC11726441

[167] De Filippis F, Pellegrini N, Vannini L, Jeffery IB, La Storia A, Laghi L, et al. High-level adherence to a Mediterranean diet beneficially impacts the gut microbiota and associated metabolome. Gut. 2016;65:1812–21. 26416813 10.1136/gutjnl-2015-309957

[168] Ghosh TS, Rampelli S, Jeffery IB, Santoro A, Neto M, Capri M, et al. Mediterranean diet intervention alters the gut microbiome in older people reducing frailty and improving health status: the NU-AGE 1-year dietary intervention across five European countries. Gut. 2020;69:1218–28. 32066625 10.1136/gutjnl-2019-319654 PMC7306987

[169] Marseglia A, Xu W, Fratiglioni L, Fabbri C, Berendsen AAM, Bialecka-Debek A, et al. Effect of the NU-AGE diet on cognitive functioning in older adults: a randomized controlled trial. Front Physiol. 2018;9:349. 29670545 10.3389/fphys.2018.00349 PMC5893841

[170] Xu Z, Knight R. Dietary effects on human gut microbiome diversity. Br J Nutr. 2015;113(Suppl):S1-5. 25498959 10.1017/S0007114514004127 PMC4405705

[171] Tessier AJ, Wang F, Korat AA, Eliassen AH, Chavarro J, Grodstein F, et al. Optimal dietary patterns for healthy aging. Nat Med. 2025. 10.1038/s41591-025-03570-5. 40128348 10.1038/s41591-025-03570-5 PMC12092270

[172] Di Francesco A, Deighan AG, Litichevskiy L, Chen Z, Luciano A, Robinson L, et al. Dietary restriction impacts health and lifespan of genetically diverse mice. Nature. 2024;634:684–92. 39385029 10.1038/s41586-024-08026-3 PMC11485257

[173] Litichevskiy L, Considine M, Gill J, Shandar V, Cox TO, Descamps HC, et al. Gut metagenomes reveal interactions between dietary restriction, ageing and the microbiome in genetically diverse mice. Nat Microbiol. 2025. 10.1038/s41564-025-01963-3. 40164832 10.1038/s41564-025-01963-3 PMC12360852

[174] Ruiz A, Cerdo T, Jauregui R, Pieper DH, Marcos A, Clemente A, et al. One-year calorie restriction impacts gut microbial composition but not its metabolic performance in obese adolescents. Environ Microbiol. 2017;19:1536–51. 28251782 10.1111/1462-2920.13713

[175] Pisanu S, Palmas V, Madau V, Casula E, Deledda A, Cusano R, et al. Impact of a moderately hypocaloric mediterranean diet on the gut microbiota composition of Italian obese patients. Nutrients. 2020;12:2707. 32899756 10.3390/nu12092707 PMC7551852

[176] Flanagan EW, Most J, Mey JT, Redman LM. Calorie restriction and aging in humans. Annu Rev Nutr. 2020;40:105–33. 32559388 10.1146/annurev-nutr-122319-034601 PMC9042193

[177] Severinsen MCK, Pedersen BK. Muscle-organ crosstalk: the emerging roles of myokines. Endocr Rev. 2020;41:594–609. 32393961 10.1210/endrev/bnaa016 PMC7288608

[178] Qiu Y, Fernandez-Garcia B, Lehmann HI, Li G, Kroemer G, Lopez-Otin C, et al. Exercise sustains the hallmarks of health. J Sport Health Sci. 2023;12:8–35. 36374766 10.1016/j.jshs.2022.10.003 PMC9923435

[179] Mailing LJ, Allen JM, Buford TW, Fields CJ, Woods JA. Exercise and the gut microbiome: a review of the evidence, potential mechanisms, and implications for human health. Exerc Sport Sci Rev. 2019;47:75–85. 30883471 10.1249/JES.0000000000000183

[180] Hawley JA, Forster SC, Giles EM. Exercise, the gut microbiome and gastrointestinal diseases: therapeutic impact and molecular mechanisms. Gastroenterology. 2025. 10.1053/j.gastro.2025.01.224. 39978410 10.1053/j.gastro.2025.01.224

[181] Varghese S, Rao S, Khattak A, Zamir F, Chaari A. Physical exercise and the gut microbiome: a bidirectional relationship influencing health and performance. Nutrients. 2024;16:3663. 39519496 10.3390/nu16213663 PMC11547208

[182] Lai ZL, Tseng CH, Ho HJ, Cheung CKY, Lin JY, Chen YJ, et al. Fecal microbiota transplantation confers beneficial metabolic effects of diet and exercise on diet-induced obese mice. Sci Rep. 2018;8:15625. 30353027 10.1038/s41598-018-33893-y PMC6199268

[183] Clarke SF, Murphy EF, O’Sullivan O, Lucey AJ, Humphreys M, Hogan A, et al. Exercise and associated dietary extremes impact on gut microbial diversity. Gut. 2014;63:1913–20. 25021423 10.1136/gutjnl-2013-306541

[184] Barton W, Penney NC, Cronin O, Garcia-Perez I, Molloy MG, Holmes E, et al. The microbiome of professional athletes differs from that of more sedentary subjects in composition and particularly at the functional metabolic level. Gut. 2018;67:625–33. 28360096 10.1136/gutjnl-2016-313627

[185] Bressa C, Bailen-Andrino M, Perez-Santiago J, Gonzalez-Soltero R, Perez M, Montalvo-Lominchar MG, et al. Differences in gut microbiota profile between women with active lifestyle and sedentary women. PLoS ONE. 2017;12:e0171352. 28187199 10.1371/journal.pone.0171352 PMC5302835

[186] Munukka E, Ahtiainen JP, Puigbo P, Jalkanen S, Pahkala K, Keskitalo A, et al. Six-week endurance exercise alters gut metagenome that is not reflected in systemic metabolism in over-weight women. Front Microbiol. 2018;9:2323. 30337914 10.3389/fmicb.2018.02323 PMC6178902

[187] Martin D, Bonneau M, Orfila L, Horeau M, Hazon M, Demay R, et al. Atypical gut microbial ecosystem from athletes with very high exercise capacity improves insulin sensitivity and muscle glycogen store in mice. Cell Rep. 2025;44:115448. 40154488 10.1016/j.celrep.2025.115448

[188] Erlandson KM, Liu J, Johnson R, Dillon S, Jankowski CM, Kroehl M, et al. An exercise intervention alters stool microbiota and metabolites among older, sedentary adults. Ther Adv Infect Dis. 2021;8:20499361211027067. 34262758 10.1177/20499361211027067 PMC8246564

[189] Zhu Q, Jiang S, Du G. Effects of exercise frequency on the gut microbiota in elderly individuals. Microbiol Open. 2020;9:e1053. 10.1002/mbo3.1053 PMC7424259 32356611

[190] Clauss M, Gerard P, Mosca A, Leclerc M. Interplay between exercise and gut microbiome in the context of human health and performance. Front Nutr. 2021;8:637010. 34179053 10.3389/fnut.2021.637010 PMC8222532

[191] van Wijck K, Lenaerts K, van Loon LJ, Peters WH, Buurman WA, Dejong CH. Exercise-induced splanchnic hypoperfusion results in gut dysfunction in healthy men. PLoS ONE. 2011;6:e22366. 21811592 10.1371/journal.pone.0022366 PMC3141050

[192] Karl JP, Margolis LM, Madslien EH, Murphy NE, Castellani JW, Gundersen Y, et al. Changes in intestinal microbiota composition and metabolism coincide with increased intestinal permeability in young adults under prolonged physiological stress. Am J Physiol Gastrointest Liver Physiol. 2017;312:G559–71. 28336545 10.1152/ajpgi.00066.2017

[193] Jayanama K, Theou O. Effects of probiotics and prebiotics on frailty and ageing: a narrative review. Curr Clin Pharmacol. 2020;15:183–92. 31750806 10.2174/1574884714666191120124548

[194] Ale EC, Binetti AG. Role of probiotics, prebiotics, and synbiotics in the elderly: insights into their applications. Front Microbiol. 2021;12:631254. 33584631 10.3389/fmicb.2021.631254 PMC7876055

[195] Hong CT, Chen JH, Huang TW. Probiotics treatment for Parkinson disease: a systematic review and meta-analysis of clinical trials. Aging (Albany NY). 2022;14:7014–25. 36084951 10.18632/aging.204266 PMC9512504

[196] Shokri-Mashhadi N, Navab F, Ansari S, Rouhani MH, Hajhashemy Z, Saraf-Bank S. A meta-analysis of the effect of probiotic administration on age-related sarcopenia. Food Sci Nutr. 2023;11:4975–87. 37701185 10.1002/fsn3.3515 PMC10494607

[197] Setbo E, Campbell K, O’Cuiv P, Hubbard R. Utility of probiotics for maintenance or improvement of health status in older people—a scoping review. J Nutr Health Aging. 2019;23:364–72. 30932135 10.1007/s12603-019-1187-9 PMC12280456

[198] Jukic Peladic N, Dell’Aquila G, Carrieri B, Maggio M, Cherubini A, Orlandoni P. Potential role of probiotics for inflammaging: a narrative review. Nutrients. 2021;13:2919. 34578796 10.3390/nu13092919 PMC8471548

[199] Recharla N, Choi J, Puligundla P, Park SJ, Lee HJ. Impact of probiotics on cognition and constipation in the elderly: a meta-analysis. Heliyon. 2023;9:e18306. 37539311 10.1016/j.heliyon.2023.e18306 PMC10395539

[200] Zmora N, Zilberman-Schapira G, Suez J, Mor U, Dori-Bachash M, Bashiardes S, et al. Personalized gut mucosal colonization resistance to empiric probiotics is associated with unique host and microbiome features. Cell. 2018;174:1388-1405.e1. 30193112 10.1016/j.cell.2018.08.041

[201] Salminen S, Collado MC, Endo A, Hill C, Lebeer S, Quigley EMM, et al. The International Scientific Association of Probiotics and Prebiotics (ISAPP) consensus statement on the definition and scope of postbiotics. Nat Rev Gastroenterol Hepatol. 2021;18:649–67. 33948025 10.1038/s41575-021-00440-6 PMC8387231

[202] Arnold JW, Roach J, Fabela S, Moorfield E, Ding S, Blue E, et al. The pleiotropic effects of prebiotic galacto-oligosaccharides on the aging gut. Microbiome. 2021;9:31. 33509277 10.1186/s40168-020-00980-0 PMC7845053

[203] Kadyan S, Park G, Singh P, Arjmandi B, Nagpal R. Prebiotic mechanisms of resistant starches from dietary beans and pulses on gut microbiome and metabolic health in a humanized murine model of aging. Front Nutr. 2023;10:1106463. 36824174 10.3389/fnut.2023.1106463 PMC9941547

[204] Kadyan S, Park G, Wang B, Nagpal R. Dietary fiber modulates gut microbiome and metabolome in a host sex-specific manner in a murine model of aging. Front Mol Biosci. 2023;10:1182643. 37457834 10.3389/fmolb.2023.1182643 PMC10345844

[205] Lee SH, You HS, Kang HG, Kang SS, Hyun SH. Association between altered blood parameters and gut microbiota after synbiotic intake in healthy, elderly Korean women. Nutrients. 2020;12:3112. 33053824 10.3390/nu12103112 PMC7650560

[206] Wang S, Ahmadi S, Nagpal R, Jain S, Mishra SP, Kavanagh K, et al. Lipoteichoic acid from the cell wall of a heat killed *Lactobacillus paracasei* D3–5 ameliorates aging-related leaky gut, inflammation and improves physical and cognitive functions: from *C. elegans* to mice. Geroscience. 2020;42:333–52. 31814084 10.1007/s11357-019-00137-4 PMC7031475

[207] Powell DN, Swimm A, Sonowal R, Bretin A, Gewirtz AT, Jones RM, et al. Indoles from the commensal microbiota act via the AHR and IL-10 to tune the cellular composition of the colonic epithelium during aging. Proc Natl Acad Sci U S A. 2020;117:21519–26. 32817517 10.1073/pnas.2003004117 PMC7474656

[208] Chen P, Chen F, Lei J, Zhou B. Gut microbial metabolite urolithin B attenuates intestinal immunity function in vivo in aging mice and in vitro in HT29 cells by regulating oxidative stress and inflammatory signalling. Food Funct. 2021;12:11938–55. 34747418 10.1039/d1fo02440j

[209] Kolonics A, Bori Z, Torma F, Abraham D, Feher J, Radak Z. Exercise combined with postbiotics treatment results in synergistic improvement of mitochondrial function in the brain of male transgenic mice for Alzheimer’s disease. BMC Neurosci. 2023;24:68. 38110905 10.1186/s12868-023-00836-x PMC10726509

[210] El Haddad L, Mendoza JF, Jobin C. Bacteriophage-mediated manipulations of microbiota in gastrointestinal diseases. Front Microbiol. 2022;13:1055427. 36466675 10.3389/fmicb.2022.1055427 PMC9714271

[211] Fujiki J, Schnabl B. Phage therapy: targeting intestinal bacterial microbiota for the treatment of liver diseases. JHEP Rep. 2023;5:100909. 37965159 10.1016/j.jhepr.2023.100909 PMC10641246

[212] Duan Y, Llorente C, Lang S, Brandl K, Chu H, Jiang L, et al. Bacteriophage targeting of gut bacterium attenuates alcoholic liver disease. Nature. 2019;575:505–11. 31723265 10.1038/s41586-019-1742-x PMC6872939

[213] Federici S, Kredo-Russo S, Valdes-Mas R, Kviatcovsky D, Weinstock E, Matiuhin Y, et al. Targeted suppression of human IBD-associated gut microbiota commensals by phage consortia for treatment of intestinal inflammation. Cell. 2022;185:2879-2898.e4. 35931020 10.1016/j.cell.2022.07.003

[214] Ichikawa M, Nakamoto N, Kredo-Russo S, Weinstock E, Weiner IN, Khabra E, et al. Bacteriophage therapy against pathological *Klebsiella pneumoniae* ameliorates the course of primary sclerosing cholangitis. Nat Commun. 2023;14:3261. 37277351 10.1038/s41467-023-39029-9 PMC10241881

[215] Gan L, Feng Y, Du B, Fu H, Tian Z, Xue G, et al. Bacteriophage targeting microbiota alleviates non-alcoholic fatty liver disease induced by high alcohol-producing *Klebsiella pneumoniae*. Nat Commun. 2023;14:3215. 37270557 10.1038/s41467-023-39028-w PMC10239455

[216] Gindin M, Febvre HP, Rao S, Wallace TC, Weir TL. Bacteriophage for Gastrointestinal Health (PHAGE) study: evaluating the safety and tolerability of supplemental bacteriophage consumption. J Am Coll Nutr. 2019;38:68–75. 30157383 10.1080/07315724.2018.1483783

[217] Grubb DS, Wrigley SD, Freedman KE, Wei Y, Vazquez AR, Trotter RE, et al. Phage-2 study: supplemental bacteriophages extend *Bifidobacterium animalis* subsp. *lactis* BL04 benefits on gut health and microbiota in healthy adults. Nutrients. 2020;12(Suppl):2474. 32824480 10.3390/nu12082474 PMC7468981

[218] Shen X, Wang C, Zhou X, Zhou W, Hornburg D, Wu S, et al. Nonlinear dynamics of multi-omics profiles during human aging. Nat Aging. 2024;4:1619–34. 39143318 10.1038/s43587-024-00692-2 PMC11564093

[219] Yang H, Wang T, Qian C, Wang H, Yu D, Shi M, et al. Gut microbial-derived phenylacetylglutamine accelerates host cellular senescence. Nat Aging. 2025;5:401–18. 39794469 10.1038/s43587-024-00795-w

[220] Tseng CH, Wong S, Yu J, Lee YY, Terauchi J, Lai HC, et al. Development of live biotherapeutic products: a position statement of Asia-Pacific Microbiota Consortium. Gut. 2025;74:706–13. 40011030 10.1136/gutjnl-2024-334501 PMC12013581

